# Effects of B_2_O_3_ on the Growth, Structural, and Magneto-Optical Properties of Yttrium Iron Garnet Single-Crystal Fibers

**DOI:** 10.1021/acs.cgd.5c01776

**Published:** 2026-03-04

**Authors:** Jun Young Hong, Dolendra Karki, Soumya Sridar, Paul Ohodnicki

**Affiliations:** † Department of Mechanical & Materials Science and Engineering, 110071 University of Pittsburgh, Pittsburgh, Pennsylvania 15261, United States; ‡ Department of Electrical and Computer Engineering, University of Pittsburgh, Pittsburgh, Pennsylvania 15261, United States; § Department of Physics and Astronomy, University of Pittsburgh, Pittsburgh, Pennsylvania 15261, United States; ∥ 17213 National Energy Technology Laboratory, Morgantown, West Virginia 26505, United States

## Abstract

This study explores the fabrication of yttrium iron garnet (YIG) single crystal fibers using the laser heated pedestal growth (LHPG) method with the experimental addition of B_2_O_3_. The incorporation of B_2_O_3_ facilitates the fiber fabrication process by lowering the required growth temperatures and likely modifying melt viscosity behavior, consistent with the established fluxing behavior of B_2_O_3_ and the comparative viscosity trend observed in the TMA–VFT analysis, thereby improving process efficiency while maintaining fiber quality. Structural characterization using EBSD and SC-XRD reveals a transition from polycrystalline to single-crystal behavior, with improved alignment along the [111] direction without altering the garnet structure. Magnetic measurements show increases in saturation magnetization in B_2_O_3_-assisted fibers. Three-dimensional anisotropy energy modeling, based on EBSD-derived Euler angles, indicates that the enhanced crystallinity and orientation contribute to reorientation of MCA energy distribution due to improved crystallographic alignment. Faraday rotation measurements show that the B_2_O_3_-assisted sample exhibits a rotation angle closer to reported values for high-quality YIG, suggesting improved phase purity and crystallographic quality. These findings demonstrate that B_2_O_3_-assisted LHPG growth is a scalable and nontoxic approach to producing high-performance YIG fibers for integrated photonic and magnetic field sensing applications.

## Graphical abstract

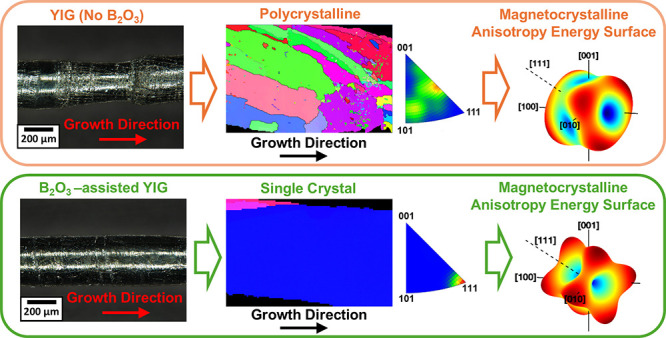

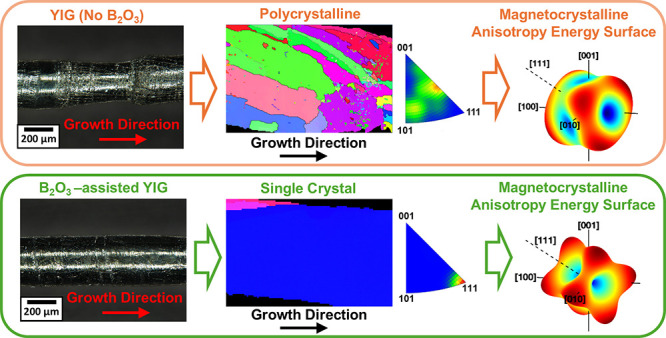

## 1 Introduction

Yttrium iron garnet (Y_3_Fe_5_O_12_, YIG) is a ferrimagnetic garnet where yttrium ions occupy dodecahedral sites and iron ions are distributed between octahedral and tetrahedral sites.^1^ This structural arrangement underpins its unique combination of magnetic and optical properties, including a high Verdet constant^2^ and low optical losses in the infrared spectrum,^3^ making YIG highly suitable for magneto-optical applications such as isolators,^4^ filters,^5^ and magnetic field sensors.^6^

In fiber form, YIG offers distinct advantages compared to bulk or film geometries. The high aspect ratio enhances Faraday rotation-based performance (θ_F_ = *VBL*, where *V* is the Verdet constant, *B* the applied magnetic field, and *L* the optical path length), providing stronger nonreciprocal effects under the same external field.^7^ This geometry also enables efficient coupling with photonic waveguides, ensuring minimal insertion loss, strong nonreciprocal phase shifts, and unidirectional light transmission. Beyond optical isolators and circulators, such properties benefit compact and lightweight systems for precision sensing, structural monitoring, and biomedical diagnostics where integration and low power operation are essential.^8–10^

The laser heated pedestal growth (LHPG) method is a promising approach for fabricating YIG single crystal fibers (SCFs) due to its crucible-free process, which minimizes contamination, and its ability to generate a steep thermal gradient and high cooling rate.^11^ These features allow the production of high-purity, small-diameter fibers with controlled crystallinity. While LHPG has been successfully applied to congruent melting materials such as sapphire and YAG,^12,13^ applying it to YIG is more challenging because of its incongruent melting behavior. The deviation from stoichiometry often results in phase separation (e.g., YFeO_3_, Fe_3_O_4_) and inclusions that degrade magneto-optical quality.^14^ Additionally, the high temperatures (>1500 °C) required for growth complicate viscosity control and melt stability, highlighting the need for process optimization.^15^

Previous studies have explored strategies to address these challenges. The floating-zone (FZ) method, for instance, has been applied to YIG but often suffers from instability due to phase separation.^16^ Process refinements such as self-adjusting solvent techniques, Fe-rich seeding, two-pass growth, and pulling rate control have improved orientation and reduced defects.^15,17^ However, these approaches remain sensitive to growth conditions and often fail to fully suppress secondary phases, a fact that points to the need for new methods.

Flux-assisted growth offers a promising solution, but many of the traditional Pb-based flux systems (e.g., PbO–PbF_2_–B_2_O_3_, PbF_2_–B_2_O_3_) present serious toxicity and volatility issues.^18,19^ Lead-free alternatives such as BaO–B_2_O_3_ ^20^ and Na_2_O–B_2_O_3_ ^21^ have been investigated, yet these require strict compositional control or suffer from narrow crystallization windows, often resulting in nonuniform or clustered microstructures. Multicomponent lead-free flux systems such as BaO–B_2_O_3_–BaF_2_ ^22^ have been demonstrated in solution-based growth. However, in LHPG the small, free-standing molten zone can be sensitive to compositional drift during pulling; therefore, we selected B_2_O_3_ as a single-component fluxing additive to reduce molten-zone compositional complexity, which can reduce contamination risks and suppress unwanted secondary phases that compete with YIG crystallization. Additionally, B_2_O_3_ is reported to expand the primary crystallization field for YIG by enhancing Fe_2_O_3_ solubility.^23^ Importantly, boron is reported to remain in the flux phase as (BO_3_)^3–^ defect complexes rather than substituting directly into the YIG lattice, thus modifying melt properties without altering the intrinsic garnet structure.^23–25^

In this study, the addition of B_2_O_3_ as a fluxing agent for LHPG growth of YIG SCFs is investigated. Although surface morphology may exhibit striation-like features, the term ‘single-crystal’ is justified here on EBSD phase mapping (>99% YIG phase with dominant [111] orientation) and SC-XRD refinement, which confirm high crystallographic quality. Structural characterization shows that B_2_O_3_ addition improves crystallographic alignment while suppressing unwanted phases. Magnetic and magneto-optical characterization demonstrates that B_2_O_3_-assisted fibers exhibit enhanced performance, with Faraday rotation angles approaching values reported for high-quality YIG. Furthermore, EBSD-derived orientations are integrated into three-dimensional magnetic energy modeling to evaluate how improved crystallinity influences the balance between magnetocrystalline anisotropy (MCA) and shape anisotropy (SA), providing direct comparison with experimental magnetization data. Collectively, these results highlight B_2_O_3_-assisted LHPG as a scalable, lead-free route for producing high-performance YIG fibers suitable for integrated photonic and magnetic sensing applications.

## 2 Experimental Methods

### 2.1 Sample Preparation

YIG and B_2_O_3_-doped YIG source pellets were prepared by mixing Fe_2_O_3_ and Y_2_O_3_ powders (3:5 molar ratio) with 0.5, 1, or 5 wt % B_2_O_3_, followed by high-energy ball milling in ethanol for 9 h at 300 rpm. The dried powders were pressed into pellets and sintered at 1400 °C for 12 h. Rectangular feed rods (≈800 × 800 μm cross-section, 2–3 cm length) were cut and polished for the fiber growth in LHPG. Fibers were grown using a CO_2_-laser based LHPG system equipped with active laser power feedback control to maintain stable molten zone conditions^26^ (a schematic is provided in the Supporting Information, Figure S1). The pulling rate was fixed at 0.2 mm/min with a target fiber diameter of ∼330 μm, monitored in real time by orthogonal cameras. A 200 μm diameter Pt wire was used to draw the fiber from the molten zone, with diameter feedback actively adjusting laser power during growth to maintain uniform fiber dimensions.

### 2.2 Sample Characterization

The microstructure of sintered pellets was examined by field emission scanning electron microscopy (FESEM, Zeiss Sigma 500 VP) at 2 kV. Elemental compositions were analyzed using energy dispersive spectroscopy (EDS, Oxford Aztec X-EDS) attached to the FESEM. Phase identification was performed by powder X-ray diffraction (PXRD, Bruker D8, Cu Kα, λ = 1.5460 Å) in the 2θ range 10–70° (step size 0.02°), using X’Pert HighScore Plus with the ICSD database.

Thermal behavior was investigated by differential thermal analysis (DTA, NETZSCH DSC 404 F1) to determine liquidus (*T*_L_) and solidus (*T*_S_) temperatures, heating from room temperature to 1550 °C at 5 °C/min in alumina crucibles. Viscosity was measured by a thermomechanical analyzer (TMA, NETZSCH 402 F1) using the parallel plate method^27^ with a constant 0.02 N load, 5 °C/min heating rate, and alumina plates.

Viscosity (η) was calculated as^28^

1 $$\eta =\frac{2\pi Fd^{5}}{3V\left(dd/dt\right)\;\left(2\pi d^{3}+V\right)}$$

where *F* is applied force, *d* is specimen height, *V* is specimen volume, and *t* is time. Data were fitted to the Vogel–Fulcher–Tammann (VFT) equation

2 $$\eta \left(T\right)=\eta_{0}\times \exp\left(\frac{B}{T-T_{0}}\right)$$

where η­(*T*) is the viscosity at temperature *T*, and η_0_, *B*, *T*_0_ are temperature-independent constants for the given system.

Single-crystal XRD (SC-XRD) was performed on LHPG-grown fibers using a Bruker D8 VENTURE with IμS 3.0 Mo Kα source. Data were indexed using APEX4, with space group determination and absorption correction via XPREP. Fiber surfaces were examined using a Zeiss Smartzoom 5 Digital Microscope before EDS mapping and Electron Backscatter Diffraction (EBSD, FEI Apreo SEM, EDAX detector). Fibers were mounted along the growth axis (*z*-axis) and polished to prepare longitudinal sections through the center, followed by colloidal silica finishing. EBSD was acquired at 15 kV, 15 mm working distance, and 70° tilt; orientation distribution function (ODF) data (5° step) were obtained for magnetocrystalline anisotropy (MCA) modeling.

Magnetic hysteresis (*M*–*H*) loops were measured using a LakeShore 8600 VSM at room temperature with the applied field parallel to the fiber growth axis with a maximum field of ±5 kOe. Full hysteresis loops were collected with a 1 Oe field step, while the low-field region (|*H*| ≤ 10 Oe) was additionally measured with a finer 0.1 Oe step to improve resolution near coercivity. Saturation magnetization (*M*_s_) was determined from the average magnetization in the high-field plateau region (|*H*| ≥ 4.5 kOe), with uncertainties given by the standard error of the mean. Remanent magnetization (*M*_r_) and coercivity (*H*_c_) were obtained from linear least-squares fits to the low-field region (|*H*| ≤ 10 Oe). M_r_ was taken from the intercept at *H* = 0, while *H*_c_ was calculated from the zero-magnetization intercepts of each hysteresis branch, averaged as (|*H*_c_^+^|+|*H*_c_^–^|)/2. Uncertainties for *M*_r_ and *H*_c_ were propagated from the standard errors of the fitted slope and intercept and combined in quadrature when averaging the two branches.

High-temperature *M*–*T* measurements (300–900 K) were performed in the same parallel geometry, with the fiber growth axis parallel to the applied magnetic field. A constant 5 kOe magnetic field was applied, parallel to the fiber axis, at a ramp rate of 5 K/min, with data collected every 15 K and averaged over 0.1 s to reduce noise. The fiber was secured in a nonmagnetic ceramic fixture within the high-temperature furnace holder to ensure mechanical stability and thermal contact.

Angular-dependent *M*–φ loops were recorded at 100–150 Oe in 1° steps; the fiber axis was mounted perpendicular to the vertical rotation axis and rotated about the vertical axis relative to the fixed field direction. *M*–*H* loops were then measured at *M*_max_ and *M*_min_ angles to calculate ΔArea.

## 3 Results and Discussion

### 3.1 Analysis of Sintered YIG and B_2_O_3_ Doped YIG Pellet

Figure 1a shows the PXRD patterns of sintered YIG and B_2_O_3_-doped YIG pellets (0.5, 1, and 5 wt %). The undoped and moderately B_2_O_3_-doped compositions (0.5 and 1 wt %) primarily exhibit the cubic YIG phase, with minor residual Fe_2_O_3_ phases. The corresponding phase fraction analysis in Figure 1b indicates high YIG phase purity (∼99.2%) for 0.5 and 1 wt % B_2_O_3_-doped YIG pellets, compared with ∼98.2% for the undoped YIG pellet. In contrast, the 5 wt % B_2_O_3_-doped pellet shows the emergence of a secondary YBO_3_ phase and an increased Fe_2_O_3_ phase fraction, suggesting oversaturation associated with excessive B_2_O_3_ addition. Supporting SEM micrographs of the pellet surfaces are provided in the Supporting Information (Figure S2) and show that moderate B_2_O_3_ additions promote more uniform grain growth consistent with a transient liquid-phase-assisted sintering effect. Similar microstructural effects, including densification and grain growth, have been reported for BaO–B_2_O_3_–BaF_2_ flux systems.^22^ Overall, these results indicate that B_2_O_3_ improves microstructure and phase purity at moderate levels (0.5–1 wt %), whereas excessive addition (5 wt %) leads to a coarser pellet surface morphology and increased secondary phase formation.

**Figure 1:**
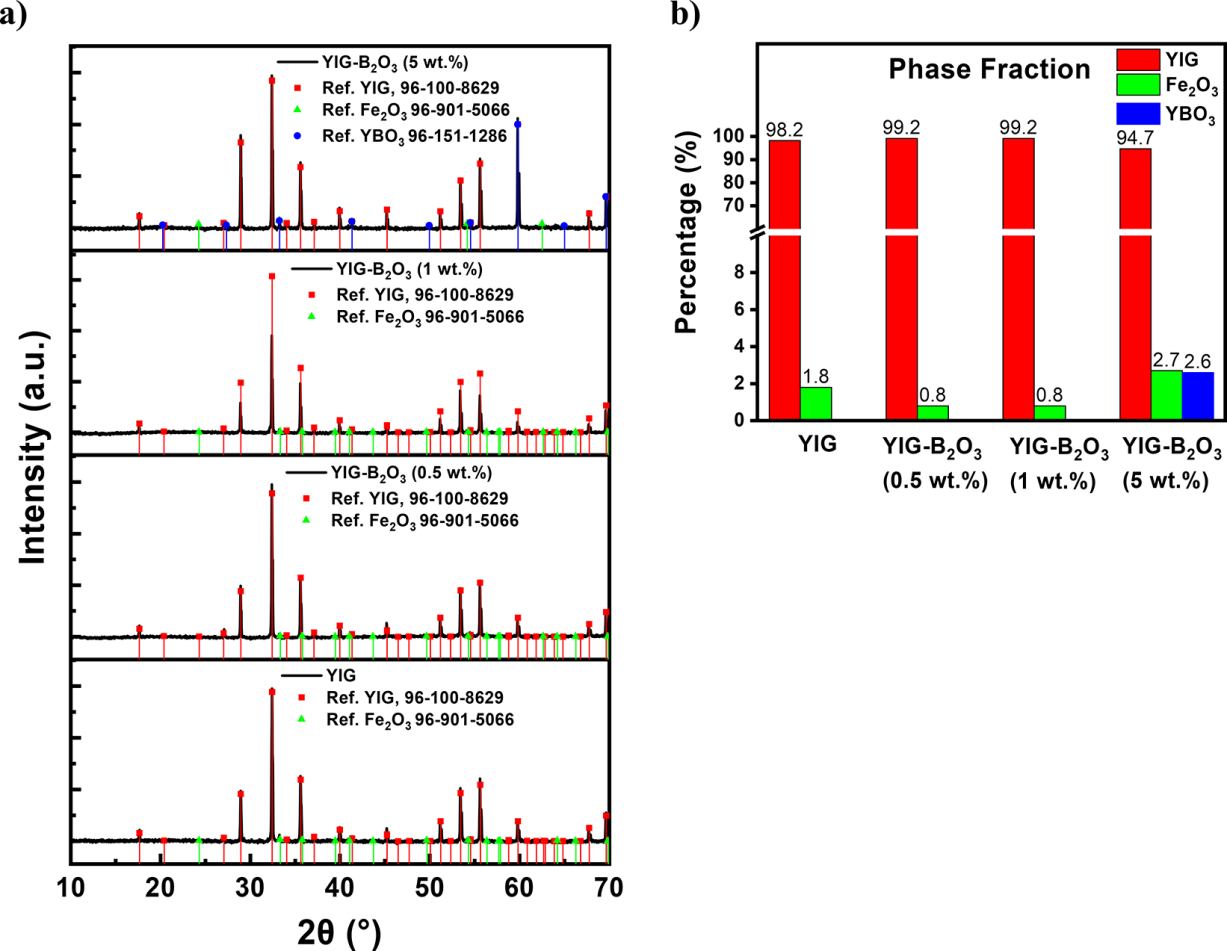
(a) PXRD patterns and corresponding (b) phase fractions of sintered YIG and B_2_O_3_-doped YIG pellets.

The impact of B_2_O_3_ doping on the thermal and rheological properties of YIG is presented in Figure 2. Differential thermal analysis (DTA) curves show a systematic decrease in the solidus temperature (*T*_S_) with increasing B_2_O_3_ content, while the liquidus temperature (*T*_L_) remains approximately constant as shown in Figure 2a. For YIG, T_S_ is 1459.2 °C, but this decreases to 1367.5 °C for YIG–B_2_O_3_(5 wt %). This reduction in *T*_S_ indicates that B_2_O_3_ facilitates earlier melting and liquid phase formation during the LHPG process. The approximately constant *T*_L_ across doping levels, within the experimental error of ±4–7 °C (accounting for instrumental precision, peak detection uncertainty, and baseline drift), suggests that the primary YIG phase remains thermally stable.

**Figure 2:**
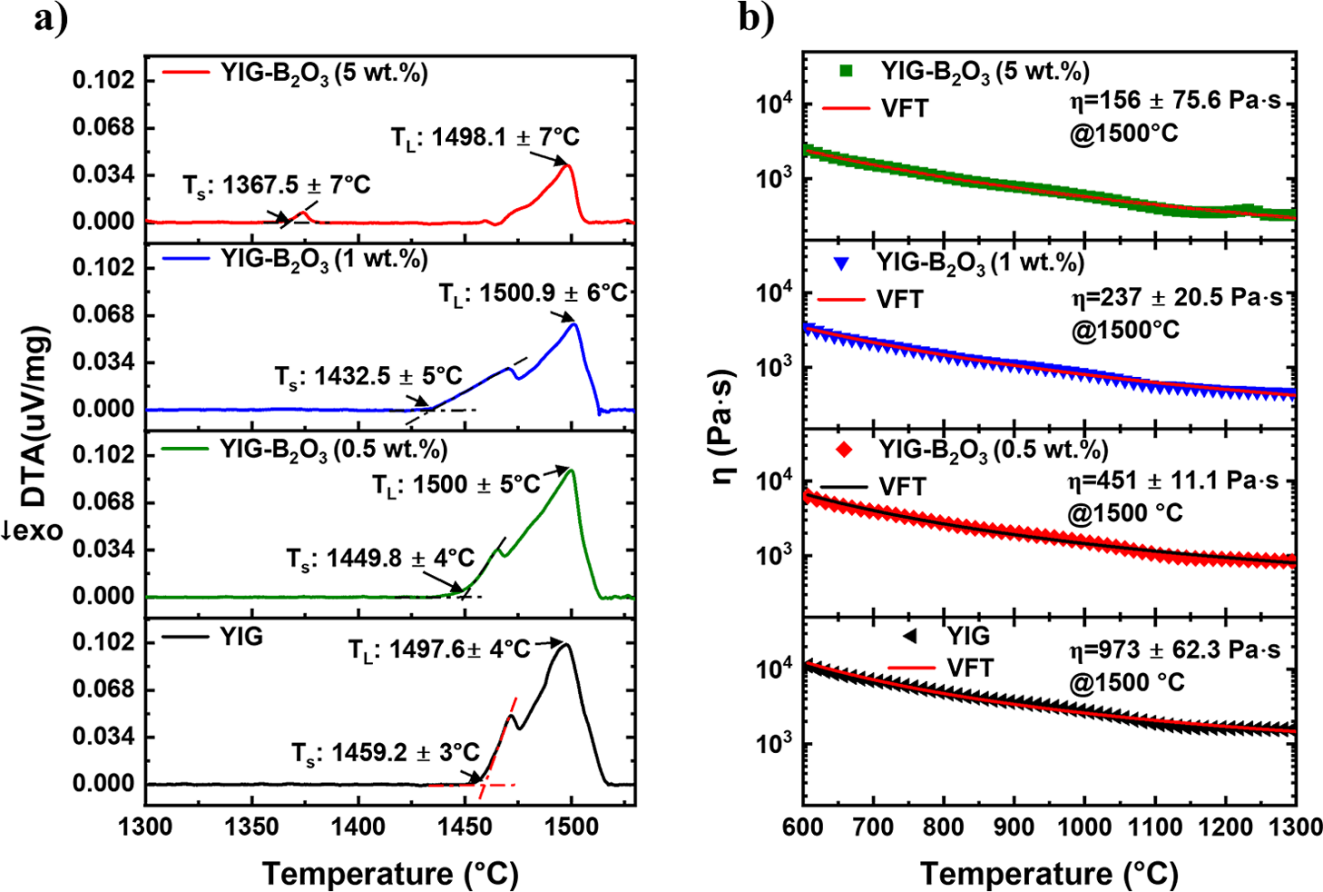
(a) DTA curves showing solidus and liquidus temperatures. Error bars account for instrumental precision, peak detection uncertainty, and baseline drift. (b) Temperature-dependent viscosity of YIG and B_2_O_3_-doped YIG pellets fitted using VFT model. Error bars represent 1σ (standard deviation) from multiple VFT fittings.

Figure 2b shows the temperature-dependent viscosity (η) fitted using the Vogel–Fulcher–Tammann (VFT) model. At 1500 °C the viscosity decreases from 973 ± 62.3 Pa·s for YIG to 156 ± 75.6 Pa·s for YIG–B_2_O_3_ (5 wt %). It is important to note that this reduction is inferred from the VFT fitting, as viscosity was not directly measured at 1500 °C. Reduction in viscosity reflects enhanced material flow within the molten zone, facilitating stable molten zone formation during LHPG. This finding aligns with the study by Sinn,^29^ which measured shear viscosity in garnet high-temperature solutions and found that addition of B_2_O_3_ significantly reduces viscosity by lowering the activation energy for viscous flow. Sinn observed that systems such as PbO–B_2_O_3_ exhibit a linear relationship between logarithm of viscosity and reciprocal temperature, further confirming the fluxing effect of B_2_O_3_. Therefore, the reduced viscosity in B_2_O_3_-doped YIG indicates lower energy barriers for atomic transport and improved molten zone control during LHPG.

The molten zone stability during LHPG was analyzed using image processing techniques to quantify its shape and size, as shown in Figure 3b. Frames were extracted from LHPG growth videos and thresholded to isolate the molten zone. The aspect ratio, representing the symmetry and stability of the molten zone, was determined based on the bounding box dimensions of the segmented region, while the molten zone area was calculated by pixel counting within the defined threshold. To ensure measurement accuracy, images were captured using a fixed optical setup, and pedestal control was maintained to minimize misalignment effects. Additionally, the feedstock was carefully centered before each experiment to ensure consistent positioning. Postprocessing steps, including bounding box normalization and contour selection, were applied to enhance measurement reliability. Measurements were averaged across two cameras, and discrepancies between them were used to quantify uncertainty, ensuring robust and repeatable results. These analyses provide a reliable framework for evaluating influence of B_2_O_3_ doping on molten zone properties.

**Figure 3:**
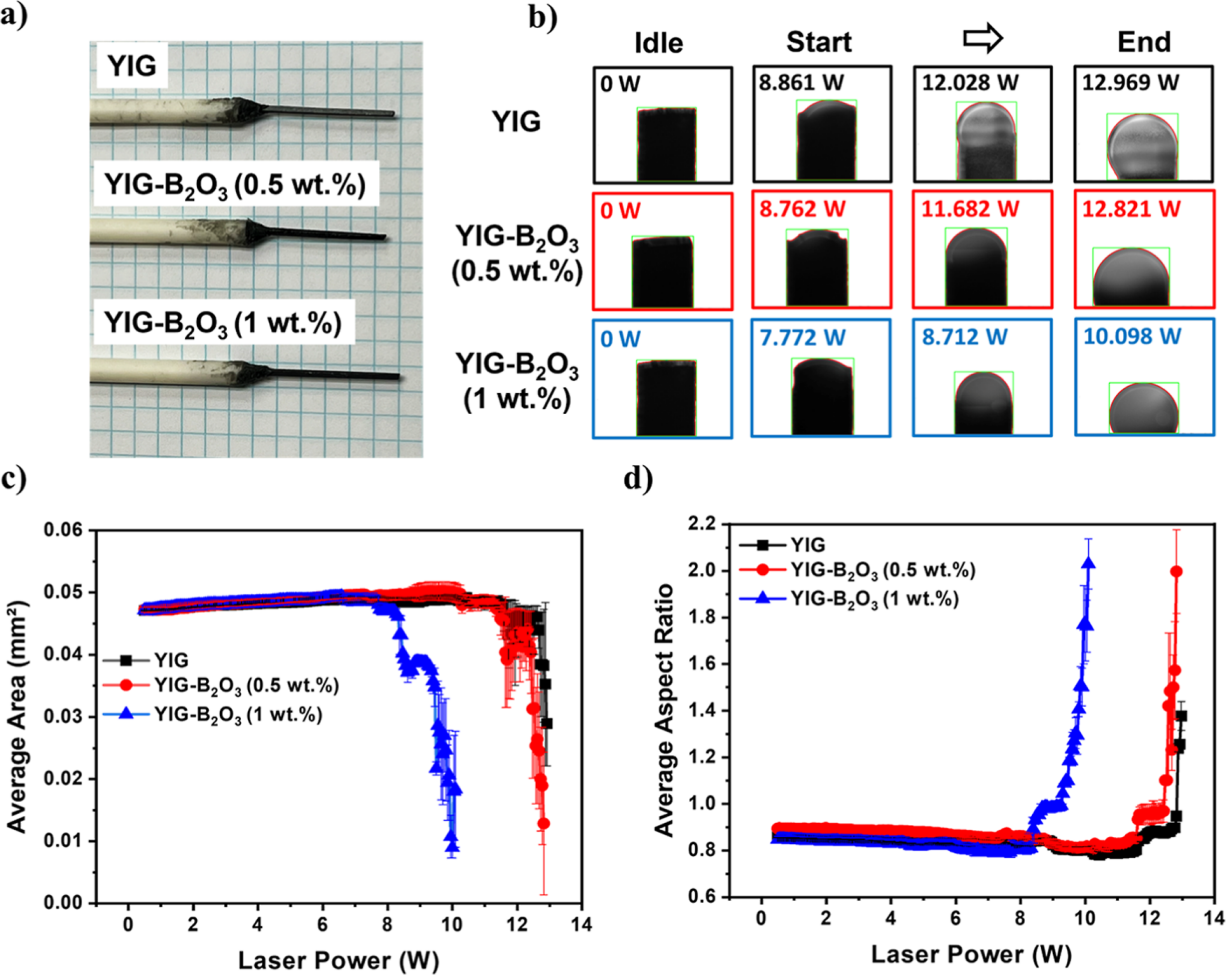
(a) Diced YIG and B_2_O_3_-doped YIG pellet samples mounted on alumina rods for LHPG processing, (b) molten zone formation under increasing laser power, (c) average molten zone area changes, and (d) aspect ratio analysis as a function of laser power.

As shown in Figure 3a, all sintered YIG and B_2_O_3_-doped YIG pellets were diced and polished to uniform dimensions. This controlled geometry ensured consistent heat transfer from the laser focal point to the molten zone and maintained identical nominal starting conditions across samples. This step minimizes geometric variations, ensuring that observed differences in molten zone dynamics arise primarily from intrinsic material properties rather than sample inconsistencies.

In Figure 3d, the aspect ratio quantifies the symmetry of the molten zone. Ideal spherical zones have an aspect ratio close to 1, indicating stability. The results show that B_2_O_3_-doped samples maintain aspect ratios closer to 1 at lower laser power that confirms improved stability. For instance, YIG–B_2_O_3_(1 wt %) achieves an aspect ratio near 1 at 9.306 W, YIG–B_2_O_3_(0.5 wt %) at 12.461 W, while YIG requires 12.864 W to reach similar stability. Figure 3c presents the molten zone area as a function of laser power. Area calculations reveal that the area drops as laser power increases which shows the onset of drastic area changes corresponding to molten zone formation. The required laser power for this transition differs depending on the B_2_O_3_ content. For example, YIG–B_2_O_3_(1 wt %) exhibits a drastic drop in area which indicates the initiation of molten zone formation at 7.785 W, while YIG–B_2_O_3_(0.5 wt %) requires 10.251 W and YIG requires 11.616 W to reach a similar initiation point.

The combination of area and aspect ratio analyses reveals insights into molten zone dynamics during LHPG. The aspect ratio confirms the symmetry and stability of the molten zone, with higher symmetry reflecting better control over the growth process. On the other hand, the area changes provide information about the onset and progression of molten zone formation as the laser power increases. Together, these analyses show that B_2_O_3_ doping reduces the required laser power for molten zone formation and stabilizes the zone by maintaining a spherical shape. This interplay of size and shape control highlights the role of B_2_O_3_ in improving the efficiency and precision of molten zone growth that directly supports the observations from DTA and viscosity analyses in Figure 2, where the reduced *T*_S_ and viscosity enable molten zone formation at lower laser power and smoother growth conditions.

### 3.2 Analysis of YIG and B_2_O_3_-Assisted YIG Crystal

Figure 4 illustrates the effect of B_2_O_3_ addition on the uniformity, surface quality, and growth stability of YIG crystals during the laser-heated pedestal growth (LHPG) process. Figure 4a provides optical microscope images of YIG, YIG–B_2_O_3_(0.5 wt %), and YIG–B_2_O_3_(1 wt %) crystals, showing variations in diameter and surface features along the growth axis. In the YIG sample (without B_2_O_3_), significant diameter fluctuations and surface irregularities were observed, particularly in areas 1, 2, and 3. The surface of the YIG crystal exhibits numerous defects such as strips, to which instability in the molten zone during growth contributes.

**Figure 4:**
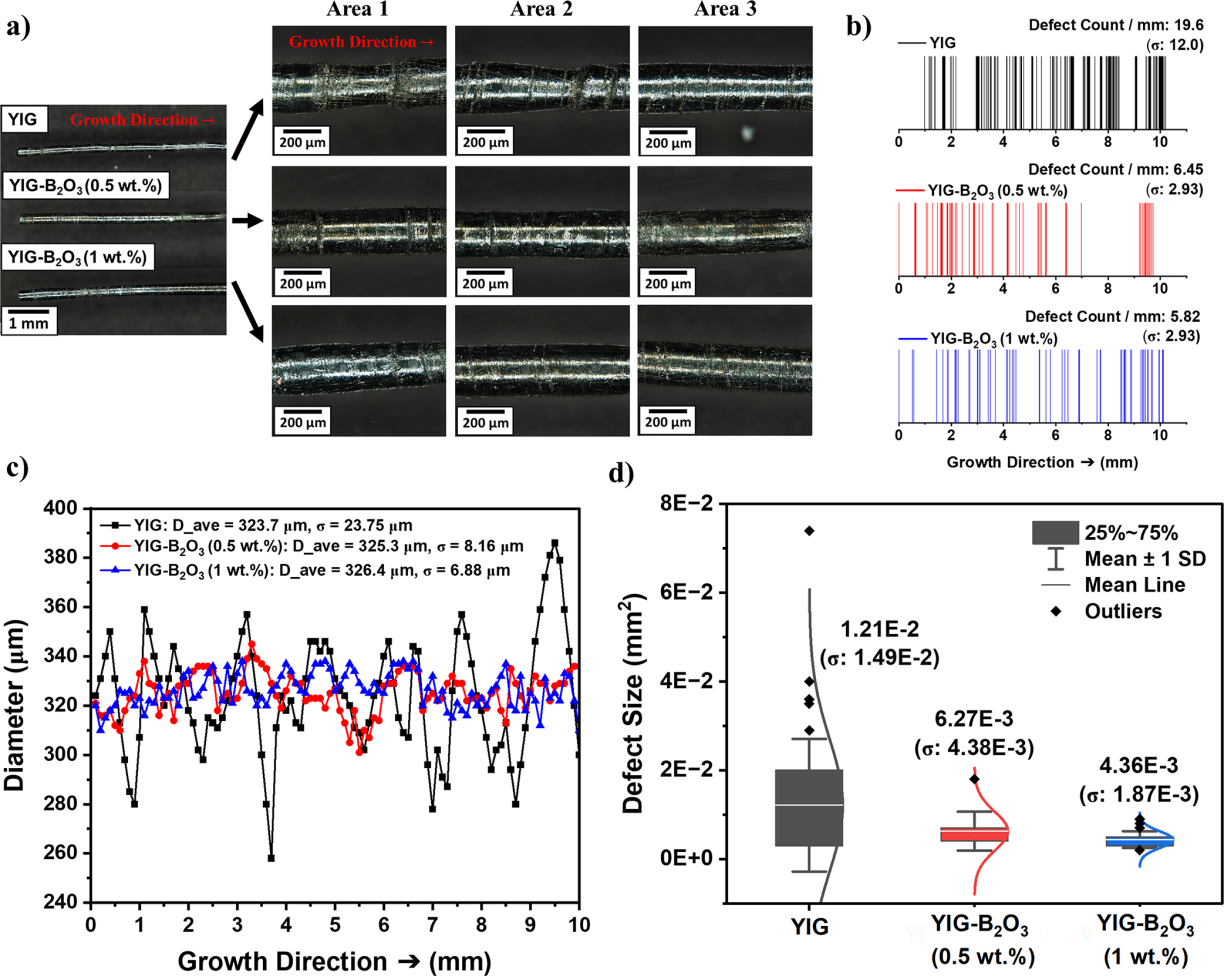
(a) Optical microscope images showing representative regions (areas 1, 2, and 3) (b) defect count per millimeter and its spatial distribution along the fiber length (c) diameter variations along the growth direction (d) defect size distribution of YIG and B_2_O_3_-assisted YIG samples.

With the addition of B_2_O_3_, these irregularities were progressively reduced. In this study, these surface irregularities–hereafter referred to as “defects”–are defined as visible morphological features on the fiber surface with a width of at least 200 μm along the fiber diameter and a length of at least 50 μm along the growth direction, as observed under an optical microscope. These defects include surface striations, ridges, and diameter discontinuities that are likely caused by molten zone instability, compositional fluctuations, or laser power variations during the LHPG process.^30^ Figure 4b quantifies the defect density, showing a high count in YIG (19.6 defects/mm, σ = 12.0) compared to 6.45 defects/mm (σ = 2.93) for YIG–B_2_O_3_(0.5 wt %) and 5.82 defects/mm (σ = 2.93) for YIG–B_2_O_3_(1 wt %), highlighting the defect suppression effect of B_2_O_3_. Figure 4c demonstrates this trend as well, where YIG exhibits significant diameter fluctuations (*D*_avg_ = 323.7 μm, σ = 23.75 μm), while YIG–B_2_O_3_(0.5 wt %) (*D*_avg_ = 325.3 μm, σ = 8.16 μm) and YIG–B_2_O_3_(1 wt %) (*D*_avg_ = 326.4 μm, σ = 6.88 μm) show reduced variation.

Figure 4d further reinforces this trend, showing B_2_O_3_ doping reduces not only defect count but also defect size. The average defect size for YIG is 1.21 × 10^–2^ mm^2^ (σ = 1.49 × 10^–2^ mm^2^), whereas YIG–B_2_O_3_(0.5 wt %) and YIG–B_2_O_3_(1 wt %) exhibit significantly smaller defects at 6.27 × 10^–3^ mm^2^ (σ = 4.38 × 10^–3^mm^2^) and 4.36 × 10^–3^ mm^2^ (σ = 1.87 × 10^–3^mm^2^), respectively. These improvements highlight the role of B_2_O_3_ in stabilizing growth, reducing dimensional variation and enhancing surface quality.

Time-resolved traces of laser power and fiber diameter (Supporting Information, Figure S3) further support the relationship between process stability and diameter control during LHPG. The pulling rate was fixed at 0.2 mm/min, and PID control coefficients (proportional, integral, and derivative gains) were kept identical across all samples to ensure consistent growth conditions. For the YIG sample, the measured diameter exhibits significant deviations from the target diameter (333 μm), fluctuating between approximately 250–450 μm, with an average diameter of 333.4 μm and a standard deviation of 27.24 μm. These fluctuations correspond to unstable laser power variations (average 11.3 W, σ = 0.68 W), which disrupt the molten zone stability, leading to greater diameter irregularities and surface defects. A closed-loop feedback control system was employed to actively control the laser power in response to real-time diameter measurements, aiming to maintain a stable molten zone.^26^ However, the remaining fluctuations still contribute to the diameter instability. This indicates that higher laser power variability directly correlates with reduced process stability during LHPG growth.

In contrast, the YIG–B_2_O_3_(0.5 wt %) sample demonstrates reduced fluctuations, with an average diameter of 327.3 μm and a standard deviation of 14.32 μm. The laser power variations are also lower (average 11.01 W, σ = 0.63 W). For the YIG–B_2_O_3_(1 wt %) sample, the measured diameter aligns closely with the target (333 μm), maintaining a more consistent range with an average diameter of 332.6 μm and a significantly reduced standard deviation of 6.37 μm. This improvement corresponds to a more stable laser power profile (average 9.31 W, σ = 0.24 W), indicating that the fluxing effect of B_2_O_3_ minimizes viscosity fluctuations, thereby stabilizing the molten zone. Hence, the system requires fewer laser power adjustments to maintain a constant diameter, leading to efficient control of dimensional uniformity and a reduction in surface defects in the grown fibers.

Surface EDX mapping and compositional distribution analysis comparing YIG fibers grown without and with B_2_O_3_ addition (1 wt %) are provided in the Supporting Information (Figure S4). In the YIG (no addition of B_2_O_3_), EDX maps show prominent strip-like surface features in which the Y signal appears locally deficient relative to Fe and O. Similar Fe-rich striations and inclusions have been observed in previous studies of YIG growth, often attributed to phase segregation or incomplete formation of the garnet structure, leading to secondary phases such as YFeO_3_, Fe_3_O_4_, and Fe_2_O_3_.^14,30^ In comparison, the B_2_O_3_-assisted YIG fiber sample shows reduced prominence of these strip-like features and a more spatially consistent distribution of the measured Y and Fe signals over the examined regions (Figure S4).

Although boron (B) was included in the EDX mapping for the YIG–B_2_O_3_(1 wt %) sample in Figure S4a, its quantitative signal was consistently below the detection limit across all line-scan and spot measurements. This is consistent with the well-known difficulty of detecting light elements such as boron in EDX, due to their low X-ray yield and strong absorption of the low-energy B Kα signal. In contrast, additional EDX measurements on the surface of the source pellets prior to LHPG growth (Supporting Information Figure S5 and Table S1) confirmed the presence of B in the starting material at levels consistent with the nominal doping concentrations (0.5–5 wt %). Hence, the presence of B in the starting pellets but its absence within the detection limit of EDX in the grown fibers suggests that partial loss or redistribution of B during LHPG growth cannot be ruled out.

In the literature, B_2_O_3_ is generally considered to act as a transient fluxing agent during oxide growth: most of the boron remains in the melt as a glassy residue and is not incorporated into the crystalline lattice. Electron probe microanalysis (EPMA) measurements in prior studies^31^ have shown boron to be below the detection limit in La_2‑x_Sr_x_CuO_4_ single crystals with B_2_O_3_ addition. While B_2_O_3_ volatilization is possible, it has been shown to be negligible under typical oxidizing conditions (<1.2 wt % loss per day at 1240 °C in air^31^). However, volatilization is generally sensitive to surface area and gas atmosphere. The unique features of the LHPG process, including a small molten zone with high surface-to-volume ratio, may therefore enhance the susceptibility to redistribution or partial volatilization. Nevertheless, the exact mechanism remains to be clarified and requires further investigation.

Figure 5 presents the EBSD analysis of the cross sections of YIG and YIG–B_2_O_3_ (1 wt %) fibers, focusing on phase purity and crystallographic orientation. The corresponding quantitative values of phase fraction, mean band contrast, and mean angular deviation (MAD) are summarized in Table 1. In the YIG fiber, the Y_3_Fe_5_O_12_ phase fraction is 96.64%, with secondary phases of Fe_3_O_4_ (2.92%), Fe_2_O_3_ (0.31%), and YFeO_3_ (0.03%). These results are consistent with fiber surface EDX (Figure S4) and pellet XRD (Figure 1) where compositional variability and extra diffraction peaks indicated secondary phase formation. Some secondary phases observed in pellet XRD (e.g., YBO_3_) were not detected in the fiber EBSD results, reflecting differences introduced by remelting and resolidification during fiber growth.

**Figure 5:**
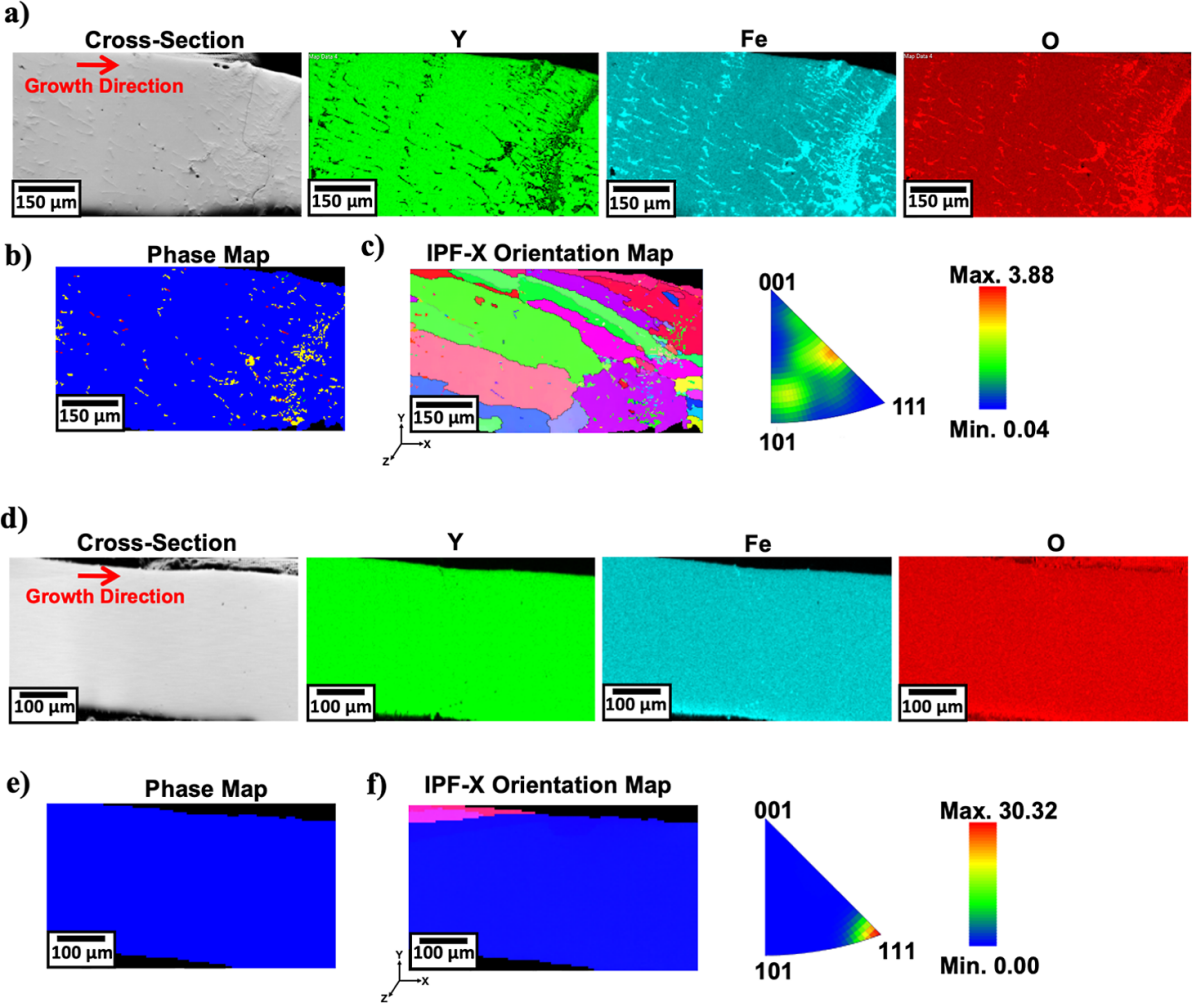
(a–c) Cross-section of YIG: (a) EDX scans, (b) phase map, and (c) IPF-X orientation map, (d–f) cross-section of YIG–B_2_O_3_ (1 wt %): (d) EDX scans, (e) phase map, and (f) IPF-X orientation map.

**Table 1:** Phase Fractions, Mean Band Contrast, and Mean Angular Deviation Values from EBSD Phase Mapping of YIG and YIG–B_2_O_3_ (1 wt.%) Fibers

phases	YIG			YIG–B_2_O_3_ (1 wt %)		
	fraction (%)	mean band contrast ± STD	mean angular deviation ± STD	fraction (%)	mean band contrast ± STD	mean angular deviation ± STD
Y_3_Fe_5_O_12_	96.64	151.6 ± 21.0	0.48 ± 0.14	99.9	159 ± 10	0.39 ± 0.11
YFeO_3_	0.03	121.2 ± 38.7	1.19 ± 0.35	0	0	0
Fe_3_O_4_	2.92	138.9 ± 21.3	0.74 ± 0.18	0	0	0
Fe_2_O_3_	0.31	135.3 ± 21.5	0.74 ± 0.18	0	0	0

For the B_2_O_3_-assisted fibers, EBSD shows an almost pure Y_3_Fe_5_O_12_ phase (99.9%) with no detectable Fe_3_O_4_, Fe_2_O_3_, or YFeO_3_. The elimination of Fe-based impurities highlights the role of B_2_O_3_ in suppressing secondary phase formation and improving phase purity during growth. While confidence in differentiating Fe_3_O_4_ and Fe_2_O_3_ is limited due to their similar MAD and band contrast values in the YIG fiber, the complete suppression of all secondary phases in the YIG–B_2_O_3_(1 wt %) fibers reinforces the observed improvement in phase purity.

Crystallographic alignment is further illustrated in the IPF-X orientation maps (Figure 5c,f). The YIG fiber exhibits diverse orientations with weak alignment (maximum intensity = 3.88), while the YIG–B_2_O_3_(1 wt %) fiber shows strong preferential alignment along the [111] axis with a maximum intensity of 30.32. This quantitative improvement demonstrates enhanced structural uniformity and directional crystallization in the presence of B_2_O_3_.

High-temperature *M*–*T* measurements (300–900 K) with the magnetic field applied parallel to the fiber axis are provided in the Supporting Information (Figure S6). The YIG fiber grown without B_2_O_3_ exhibits a broader magnetic transition near 555 K and an additional feature near ∼870 K that is consistent with Fe_3_O_4_-related contributions reported in the literature,^32^ in line with secondary-phase signals observed by EBSD (Figure 5). In contrast, fibers grown with B_2_O_3_ addition show a sharper transition near 555 K, consistent with improved homogeneity.^32^ The ∼870 K feature is also suppressed in the B_2_O_3_-assisted fibers, consistent with reduced Fe_3_O_4_-related contributions and enhanced YIG phase purity. Minor phases such as YFeO_3_ (0.03%) and Fe_2_O_3_ (0.31%) detected by EBSD (Table 1) are too scarce to produce measurable features. YFeO_3_ is weakly ferromagnetic below *T*_n_ ≈ 645 K due to Dzyaloshinskii–Moriya canting;^33^ however, given its extremely low fraction, its contribution to the bulk magnetization is insignificant. Fe_2_O_3_, with *T*_n_ ≈ 950 K,^34^ remains antiferromagnetic within our measurement window (≤900 K), and its small fraction likewise limits its effect. Therefore, the overall influence of these minor phases is negligible compared to the dominant YIG and Fe_3_O_4_ signals.

As summarized in Table 2 and Figure 6, The SC-XRD results demonstrate the effects of B_2_O_3_ addition on the structural properties of YIG crystals. The lattice parameter (*a* = *b* = *c*) expands slightly from 12.363(±0.001) Å for YIG to 12.375(±0.002) Å for YIG–B_2_O_3_ (1 wt %), and the unit cell volume correspondingly grows from 1889.5(±0.3) Å^3^ for YIG to 1894.9(±0.8) Å^3^ for YIG–B_2_O_3_ (1 wt %). While SC-XRD confirms that the cubic crystal system (*I*a3̅d) and bond angles (α = β = γ = 90°) remain unchanged, maintaining the integrity of the garnet structure. This expansion is attributed to the lattice relaxation during growth, facilitated by B_2_O_3_ acting as a fluxing agent that improves atomic mobility and reduces internal stresses.^22^

**Table 2:** Structural and Refinement Parameters From Single-Crystal XRD for YIG and YIG–B_2_O_3_ (0.5 and 1 wt.%) Samples^a^

sample	crystal system	space group	lattice parameters (*a*, *b*, *c*)	bond angle	volume (V)	*R*1; ω*R*2	goodness of fit (GooF)
ref YIG (COD:1521848)	cubic	*I*a3̅d(230)	*a* = *b* = *c* = 12.356 Å	α = β = γ = 90°	1886.5 Å^3^	NA	NA
YIG	cubic	*I*a3̅d(230)	*a* = *b* = *c* = 12.363 ± 0.001 Å	α = β = γ = 90°	1889.5 ± 0.3 Å^3^	0.153,0.265	1.45
YIG–B_2_O_3_ (0.5 wt %)	cubic	*I*a3̅d(230)	*a* = *b* = *c* = 12.368 ± 0.001 Å	α = β = γ = 90°	1892.0 ± 0.4 Å^3^	0.042,0.113	1.23
YIG–B_2_O_3_ (1 wt %)	cubic	*I*a3̅d(230)	*a* = *b* = *c* = 12.375 ± 0.002 Å	α = β = γ = 90°	1894.9 ± 0.8 Å^3^	0.031,0.107	0.93

a Expanded single-crystal XRD data collection and refinement statistics are provided in the Supporting Information (Table S2).

**Figure 6:**
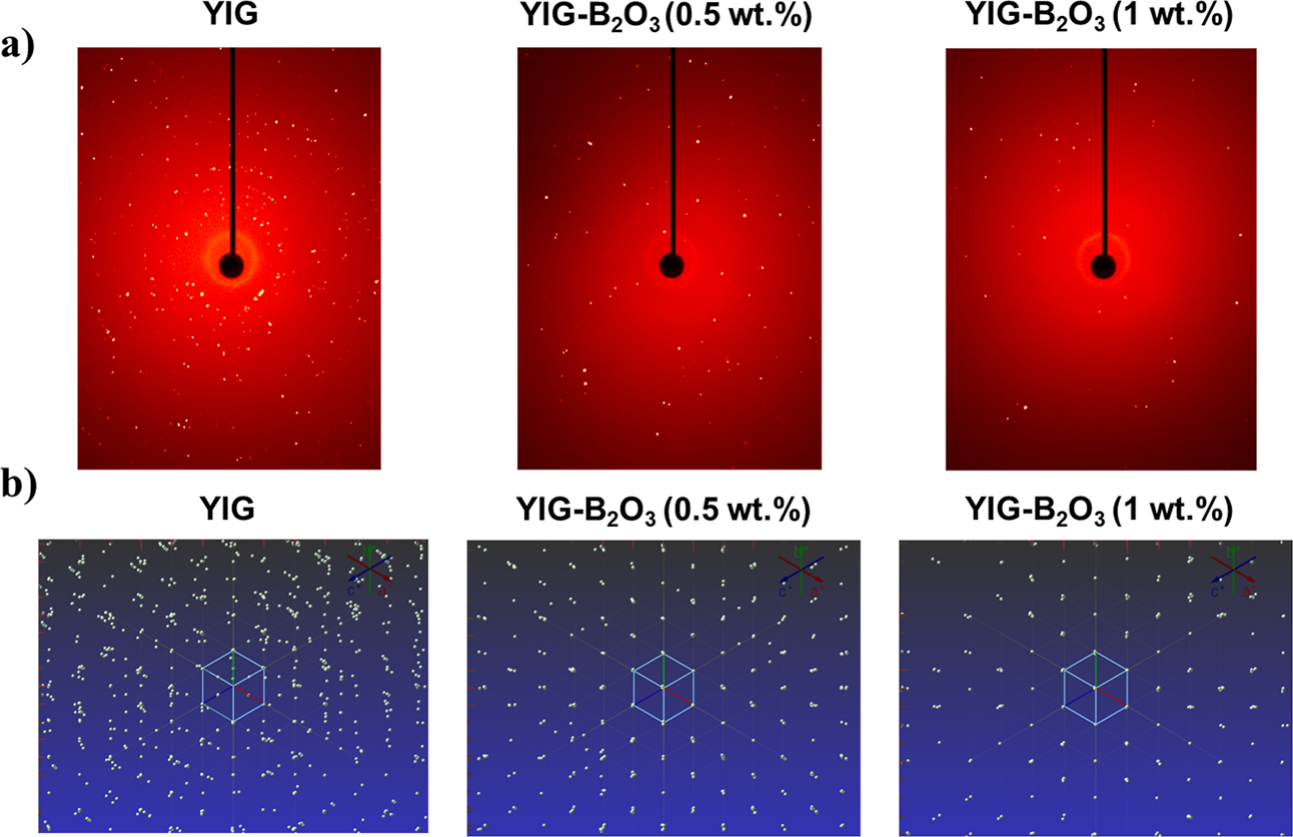
SC-XRD results for YIG and YIG–B_2_O_3_ (0.5 and 1 wt %) samples, (a) Laue patterns at a fixed goniometer orientation (2θ = 0°,Ω = Φ = 0°,χ = −35°) and (b) reciprocal lattice reconstructions visualized along the [100] axis, constructed from reflections measured across different sample orientations.

Previous studies using secondary ion mass spectrometry (SIMS) analysis of liquid-phase epitaxy (LPE)-grown YIG films have shown that boron incorporation remains minimal, with B_2_O_3_ primarily staying in the flux phase rather than substituting for Fe^3+^ or Y^3+^.^35^ These works further indicate that, when incorporated, boron tends to coordinate with oxygen rather than replace cations,^36^ forming (BO_3_)^3–^ defect complexes at interstitial positions and thereby influencing structural parameters.^24,25^ Although boron does not substitute for Y or Fe in substantial quantities, its presence in the flux phase influences crystal growth by modifying oxygen coordination environments.

Figure 6a shows the Laue patterns obtained at a fixed goniometer orientation (2θ = 0°, Ω = Φ = 0°,χ = −35°). The diffraction spots exhibit variations in sharpness and intensity, which can be attributed to differences in lattice distortions, dislocation density, and grain boundary effects. In single-crystal XRD, the full width at half-maximum (FWHM) of diffraction peaks serves as a quantitative measure of crystallinity, where sharper and more well-defined spots indicate higher structural order. In contrast, broader or diffused spots suggest the presence of defects, local strain, or subgrain boundaries. The Laue patterns in Figure 6a reveal an improvement in spot sharpness for B_2_O_3_-assisted YIG, suggesting enhanced crystallinity.

Figure 6b presents the reciprocal lattice reconstruction, generated using reflections collected from different sample orientations during SC-XRD experiments and subsequently visualized along the [100] zone axis. This reconstruction provides a comprehensive view of the crystal symmetry and structural order beyond a single diffraction pattern. While the Laue pattern in Figure 6a highlights local diffraction quality in one specific direction, the reciprocal lattice reconstruction reveals improved crystallographic alignment in B_2_O_3_-assisted YIG samples, indicating greater structural coherence and reduced lattice distortions. Although the reconstruction is visualized along [100] rather than fiber growth axis [111], the pieces of fiber were mounted without a predefined orientation, so alignment does not reflect the growth direction but rather overall structural quality.

Table 2, which presents the refinement indicators, further confirms the improved crystallographic orientation and structural coherence of B_2_O_3_-assisted YIG fibers. The refinement quality was evaluated using standard crystallographic indicators: *R*1, w*R*2, and Goodness of Fit (GooF).^37^ *R*1, the residual factor, quantifies the agreement between observed and calculated structure factor amplitudes and is defined as

3 $$R1=\frac{\sum \left|F_{0}-F_{c}\right|}{\sum F_{0}}$$

where *F*_0_ and *F*_c_ are the observed and calculated structure factors, respectively. The observed structure factor *F*_o_ is obtained from measured diffraction intensities, while the calculated structure factor *F*_c_ is determined using the refined atomic model, which incorporates atomic positions, thermal vibrations, and scattering factors. The refinement process iteratively adjusts model parameters to minimize the difference between *F*_o_ and *F*_c_, improving structural accuracy.

w*R*2, a weighted residual factor that applies additional weighting to squared structure factor differences, is given by

4 $$wR2=\sqrt{\frac{\sum w{\left(F_{0}^{2}-F_{c}^{2}\right)}^{2}}{\sum w{\left(F_{0}^{2}\right)}^{2}}}$$

where *w* represents the weighting factor. Goodness of Fit (GooF) measures how well the refinement model fits the observed data, calculated as

5 $$GooF=\sqrt{\frac{\sum w{\left(F_{0}^{2}-F_{c}^{2}\right)}^{2}}{N_{ref}-N_{par}}}$$

where *N*_ref._ is the number of reflections and *N*_par._ is the number of refined parameters.

Specifically, *R*1 values decrease significantly from 15.3% in YIG to 4.2% in YIG–B_2_O_3_ (0.5 wt %) and 3.1% in YIG–B_2_O_3_ (1 wt %), aligning with well-refined structures (*R*1 < 5% ^37^). Similarly, w*R*2 values improve from 26.5% in YIG to 11.3% in YIG–B_2_O_3_ (0.5 wt %) and 10.7% with YIG–B_2_O_3_ (1 wt %), falling within the well-refined range (w*R*2 < 12% ^37^). The Goodness of Fit (GooF) also improves from 1.45 in YIG to 1.23 in YIG–B_2_O_3_ (0.5 wt %) and 0.93 in YIG–B_2_O_3_ (1 wt %), within the ideal range (0.9–1.2 ^37^). These quantitative improvements correlate with the sharper diffraction spots and improved alignment of reciprocal lattice points observed in Figure 6 a,b, confirming enhanced structural order in the B_2_O_3_-assisted YIG samples.

In Figure 7a, the schematic shows that the M–H hysteresis loops were measured with the applied magnetic field parallel to the fiber growth axis. Representative hysteresis loops measured at room temperature are presented in Figure 7b and Table 3 show that M_s_ increases from 21.74(±0.65) emu/g for YIG to 25.13(±0.75) and 26.84(±0.80) emu/g for 0.5 and 1 wt % B_2_O_3_-assisted YIG fibers, respectively, consistent with reported values for high-quality single-crystal YIG.^38,39^

**Figure 7:**
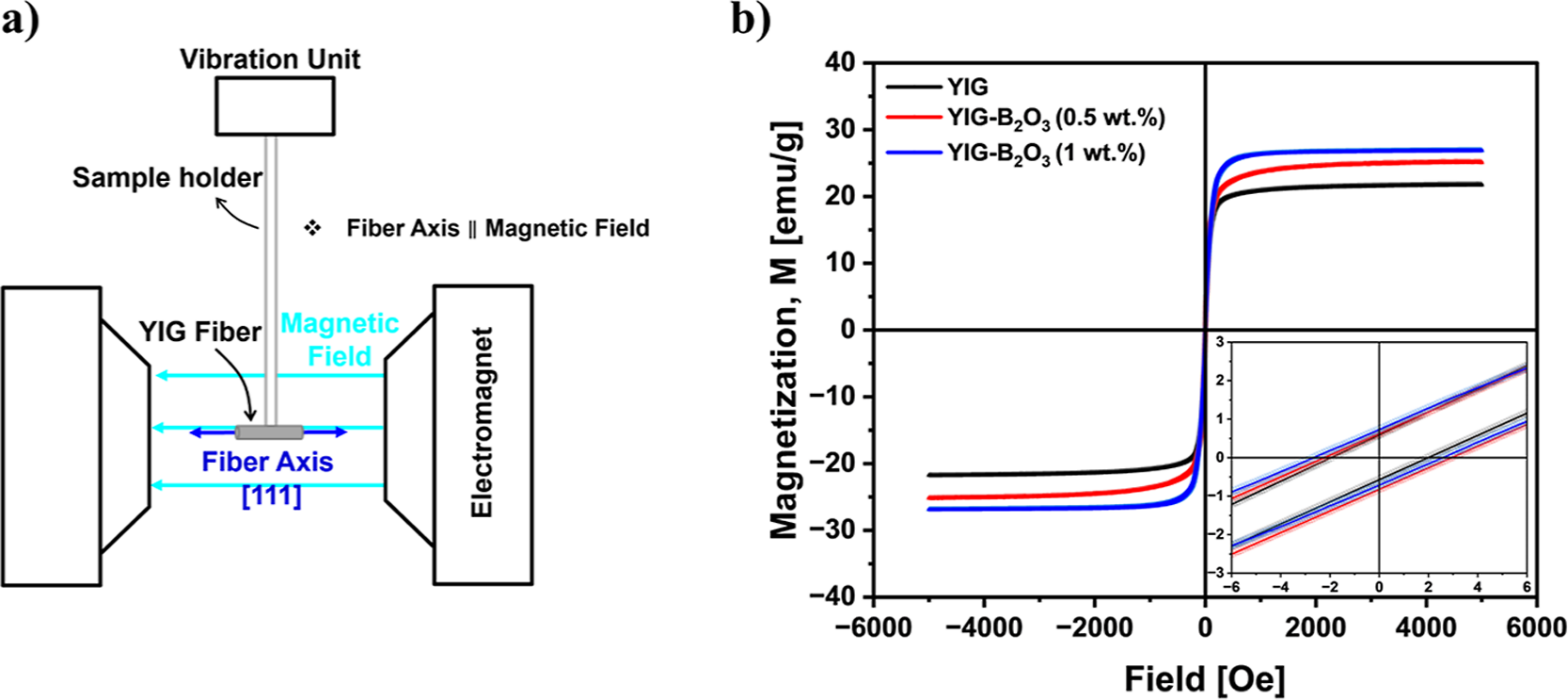
(a) Schematic of the VSM measurement geometry showing the YIG fiber mounted with its axis ([111] direction) parallel to the applied magnetic field. (b) Room-temperature hysteresis loops (*M*–*H* curves) of YIG and B_2_O_3_-assisted YIG fibers. Inset shows the expanded view of low-field region.

**Table 3:** Magnetic and Anisotropy Parameters of YIG and B_2_O_3_-Assisted YIG Samples, Including Saturation Magnetization (*M*_s_), Coercivity (*H*_c_), Remanent Magnetization (*M*_r_), Area Difference (*M*_max_ – *M*_min_) in *M*–*H* Curve, and the Amplitude of Second Harmonic (*A*_2_)­^a^

sample	*M*_s_ (emu/g)	*H*_c_ (Oe)	*M*_r_ (emu/g)	ΔArea (×10^4^ J/m^3^)	second harmonic (*A*_2_)
YIG	21.74 ± 0.65	1.97 ± 0.02	0.58 ± 0.01	2.29 ± 0.04	0.376 ± 0.003
YIG–B_2_O_3_ (0.5 wt %)	25.13 ± 0.75	2.57 ± 0.35	0.73 ± 0.01	2.67 ± 0.05	0.456 ± 0.003
YIG–B_2_O_3_ (1 wt %)	26.84 ± 0.80	2.59 ± 0.05	0.72 ± 0.01	3.40 ± 0.06	0.481 ± 0.003

a Uncertainties represent one-sigma standard errors.

In the YIG (without B_2_O_3_) fiber, the lower *M*_s_ compared to intrinsic YIG cannot be fully explained by the small fractions of secondary phases detected (Table 1), since Fe_3_O_4_ and γ-Fe_2_O_3_ have higher intrinsic magnetization than YIG. Rather, the result most likely reflects a combination of factors, including structural disorder within the YIG matrix, as suggested by EBSD (lower band contrast, higher MAD) and the broader Curie transition in dM/dT. By contrast, the B_2_O_3_-assisted YIG fibers exhibit M_s_ values much closer to intrinsic YIG, consistent with improved YIG phase purity and crystallographic quality.

Figure 7b and Table 3 show that coercivity (*H*_c_) increases from 1.97 Oe ± 0.02 Oe for YIG, 2.57 ± 0.35 for YIG–B_2_O_3_ (0.5 wt %), and 2.59 ± 0.05 Oe for YIG–B_2_O_3_ (1 wt %), while remanent magnetization (*M*_r_) is 0.58 ± 0.01, 0.73 ± 0.01, 0.72 ± 0.02 emu/g, respectively. These values were extracted from linear fits to the low-field region (|*H*| ≤ 10 Oe) of the hysteresis loops measured with 0.1 Oe steps, with uncertainties propagated from the fit parameters.

A schematic of the angular-dependent setup is shown in Figure 8a. The fiber samples were mounted with its axis perpendicular to the rotation axis (red). Angular scans were performed at 100–150 Oe, exceeding *H*_c_ but below saturation to maximize anisotropy sensitivity. Figure 8b shows the angular dependence of the normalized magnetization, with the Fourier-series fit overlaid.

**Figure 8:**
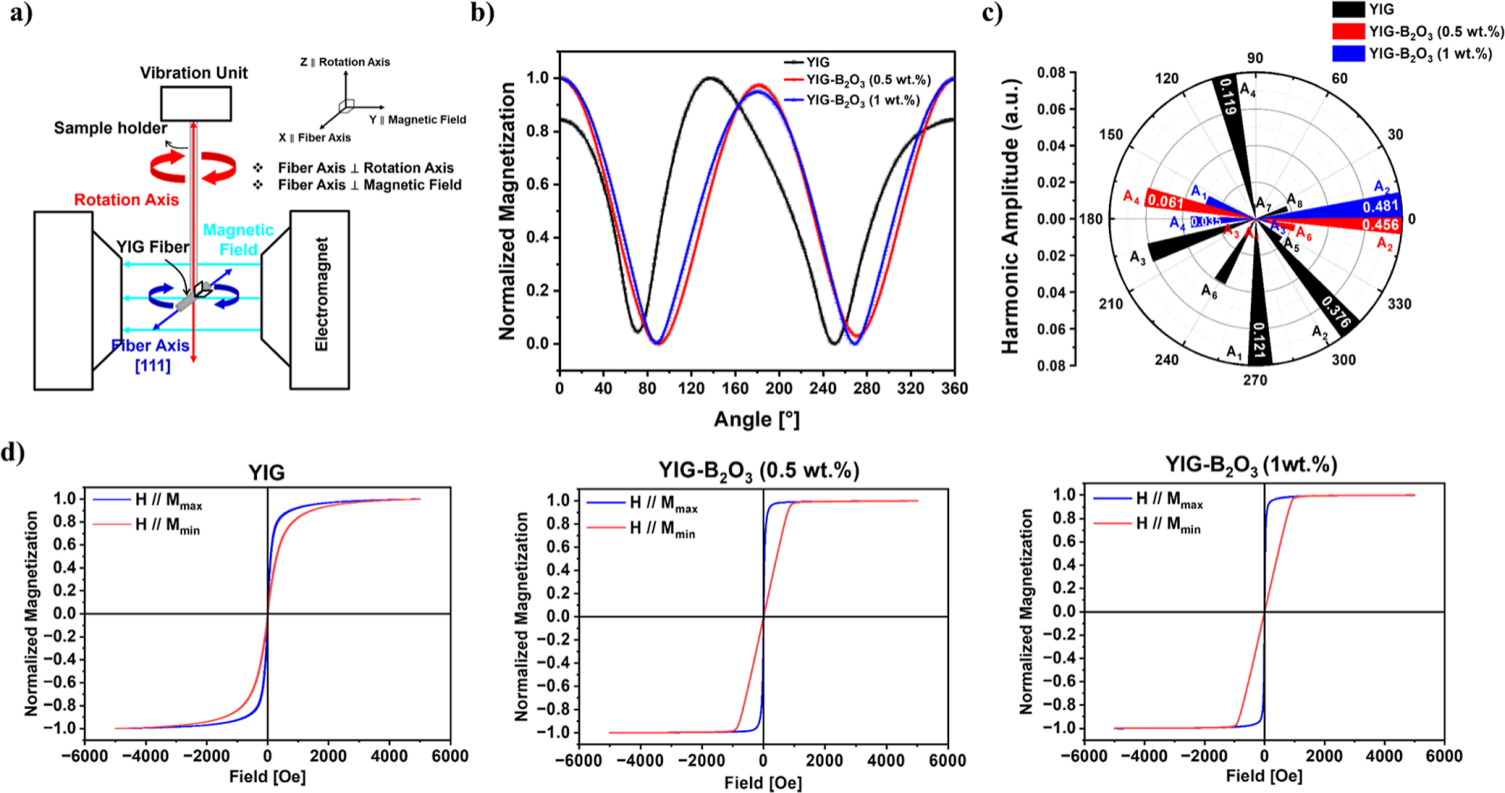
(a) Schematic of the angular-dependent magnetization measurement setup. (b) Angular dependence of normalized magnetization with Fourier fitting (lines), (c) polar plot of the amplitude of harmonic component *A*_n_ (where *n* = 1 to 8). Each sector represents the amplitude (radial length) and phase angle (angular direction) of the corresponding harmonic. (d) *M*–*H* curves measured at the angles corresponding to the maximum magnetization (*M*_max_) and minimum magnetization (*M*_min_) values observed in (b).

The polar plots in Figure 8c were obtained by fitting the angular-dependent magnetization data in Figure 8b with a Fourier series, where each amplitude $\sqrt{a_{n}^{2}+b_{n}^{2}}$ is plotted radially and its phase angle ϕ_*n*_ = arctan­(*b*_*n*_ */a*_*n*_) indicates the angular shift. In all samples, the second harmonic (2θ) dominates, consistent with the sin^2^ θ dependence of shape anisotropy. The YIG exhibits *A*_2_ = 0.376 at 314.7°, along with eight higher-order components and broader phase distribution. In contrast, the B_2_O_3_ assisted YIG fibers show suppressed higher-order terms (six for 0.5 wt % and four for 1 wt %) and stronger *A*_2_ aligned near 0° (0.456 at 358.9° for 0.5 wt % and 0.481 at 1.7° for 1 wt %). The Fourier fitting achieves *R* ^2^ ≈

99.95% with RMSE between 0.005 and 0.008, confirming reliability. These results demonstrate that B_2_O_3_ addition sharpens harmonic alignment and reduces microstructural irregularities, yielding a more uniform anisotropy landscape.

The M-H curves shown in Figure 8d are plotted for the maximum (*M*_max_) and minimum (*M*_min_) magnetization positions identified from the angular dependence graph in Figure 8b. The area differences (ΔArea) between these *M*–*H* curves are also summarized in Table 3. With increased B_2_O_3_ doping, ΔArea increases from 2.29 ± 0.04 × 10^4^ J/m^3^ for YIG to 3.40 ± 0.06 × 10^4^ J/m^3^ for YIG–B_2_O_3_(1 wt %). This trend suggests an evolution toward more pronounced directional magnetic behavior. As demonstrated in the harmonic analysis Figure 8c, the increasing second harmonic amplitude (*A*_2_) further supports this interpretation, which reflects a stronger uniaxial shape anisotropy profile.

To connect the experimentally observed crystallographic texture from EBSD with the anisotropic magnetic behavior measured by VSM, we modeled the combined magnetocrystalline anisotropy (MCA) and shape anisotropy (SA) energy surfaces using experimental inputs. This modeling framework not only integrates structural and magnetic data but also reveals how B_2_O_3_ addition influences the directional expression of anisotropy. The magnetic energy surfaces presented in Figure 10 illustrate the anisotropic magnetic behavior of YIG and YIG-B_2_O_3_(1 wt %). These surfaces were plotted to visualize the contributions of MCA and SA in a crystallographically aligned framework. More specifically, Euler angle data derived from EBSD was incorporated into the MCA calculation, directly integrating experimentally measured crystallographic information. This approach enables an accurate representation of the orientation of grains on the magnetic anisotropy. By aligning theoretical calculations with experimentally observed texture, comprehensive visualization of anisotropic magnetic behavior is possible.

**Figure 9:**
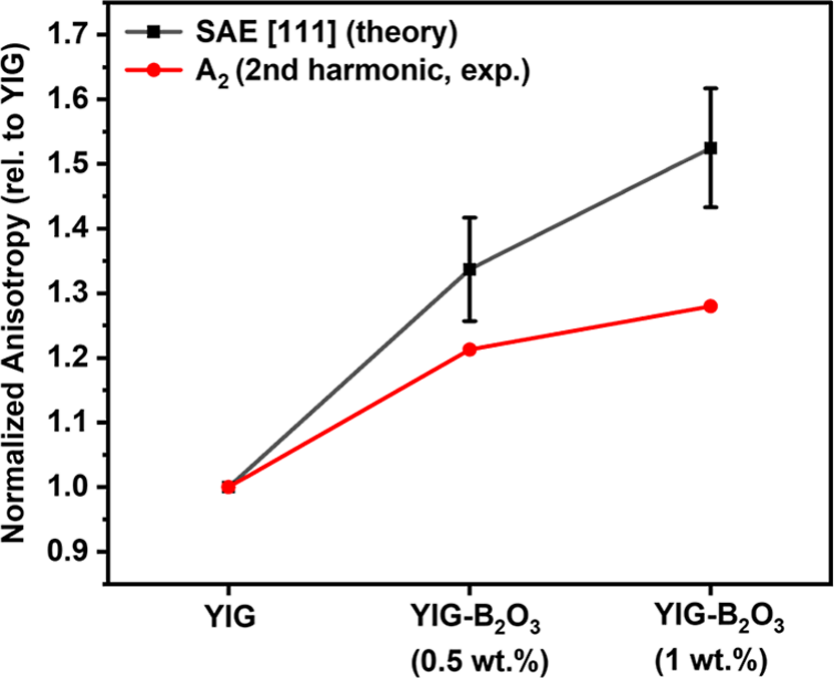
Comparison between calculated shape anisotropy energy (SAE in [111] direction) and experimentally extracted second harmonic amplitude (*A*_2_, from angular magnetization Fourier fitting as shown in Figure 8c), both normalized to YIG sample. Error bars reflect propagation from uncertainty in *M*_s_ (for SAE) and Fourier fitting (for *A*_2_).

**Figure 10:**
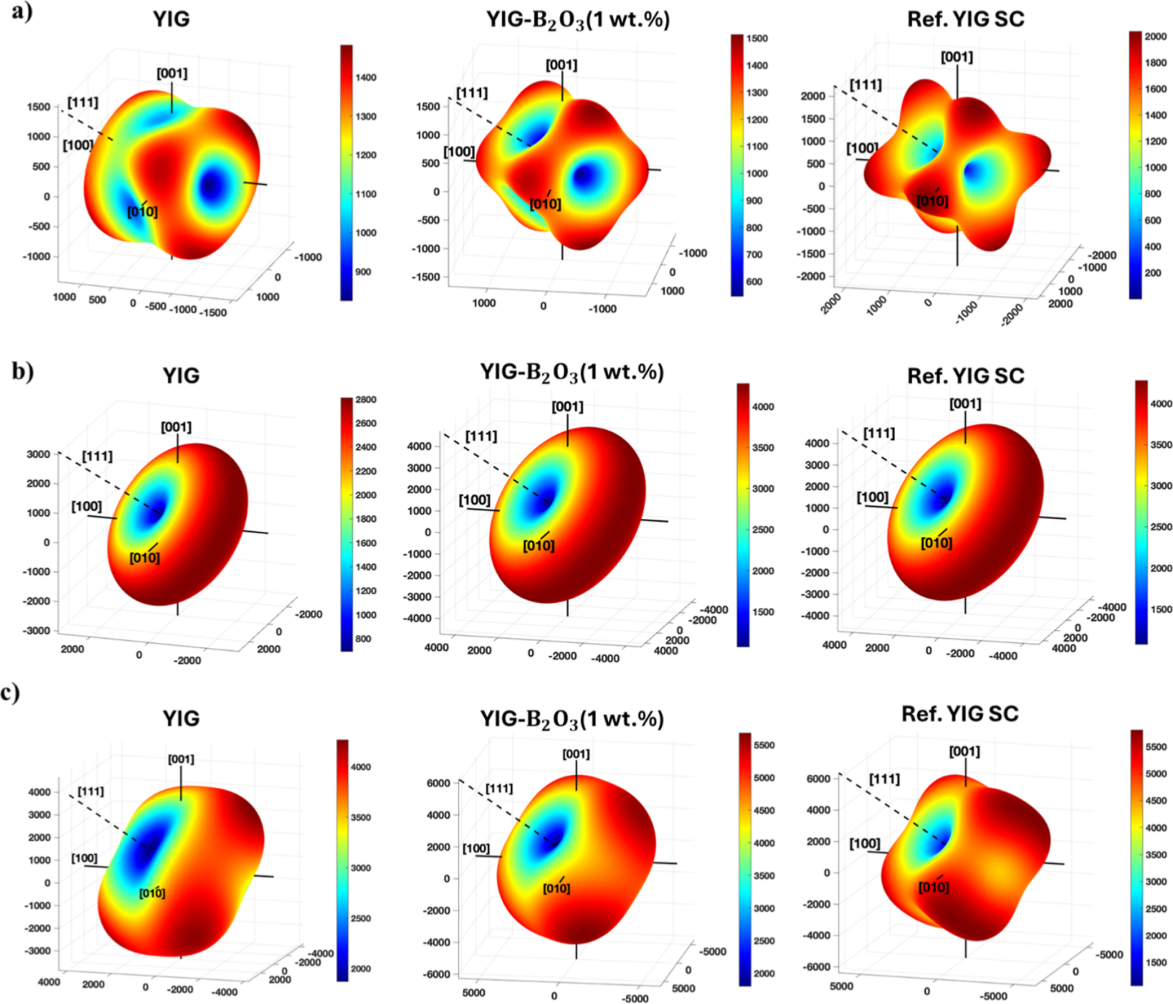
(a) Magnetocrystalline anisotropy (MCA) (b) shape anisotropy (SA) (c) combined anisotropy (MCA + SA) of YIG, 1 wt % B_2_O_3_ assisted YIG, ideal reference YIG SC samples.

The energy calculations were carried out in the spherical coordinate system, where the polar (θ) and azimuthal (ϕ) angles define spatial orientations. Separate transformations were applied to calculate MCA and SA contributions, reflecting their distinct physical origins. Both MCA and SA were formulated in the spherical coordinate system. However, their respective transformations into the crystallographic frame differ in implementation. For MCA, the *Z*–*X*–*Z* rotation convention was utilized^40^ in conjunction with Euler angles (ϕ_1_, Φ, ϕ_2_) obtained from EBSD to align the crystal frame with the experimentally determined orientations.

For SA, the calculation focused on the sample’s macroscopic geometry, with the long axis of the rod assumed to represent the direction of minimum demagnetizing energy (easy axis). To represent this axis within the crystallographic frame, a reference direction corresponding to [111] was selected based on the growth orientation in fiber samples. The *Z*–*X*–*Z* Euler rotation convention was used to transform this directional vector into the sample reference frame, applying Euler angles (ϕ_1_ = 45°, Φ = 54.74°,ϕ_2_ = 0°) corresponding to the [111] orientation in Bunge notation.^40^ This enabled projection of the shape anisotropy energy onto the same spherical coordinate system used for MCA.

For MCA, the energy was calculated using the cubic anisotropy equation with the *K*_1_ constant, excluding other terms like *K*_0_ and *K*_2_ to simplify the analysis and focus on the dominant anisotropy contribution.

6 $$U_{MCA}=K_{1}\cdot \left(\alpha_{1}^{2}\alpha_{2}^{2}+\alpha_{2}^{2}\alpha_{3}^{2}+\alpha_{3}^{2}\alpha_{1}^{2}\right)$$

where *K*_1_ value was adapted from the literature, *K*_1_ = −6100 erg/cm^3.^ ^41^ The direction cosines (α_1_, α_2_, α_3_) were transformed into the crystal frame using the *Z*–*X*–*Z* rotation matrix.

7 $$g=R_{z}\left(\phi_{2}\right)\times R_{x}\left(\Phi \right)\times R_{z}\left(\phi_{1}\right)$$

where the angles (ϕ_1_, Φ, ϕ_2_) correspond to the orientation derived from EBSD for [111]-aligned grains. This approach provides a direct link between crystallographic data and the modeled MCA energy surface.

For SA, the energy was calculated using the following equation

8 $$U_{SA}=\frac{1}{2}M_{s}^{2}\cdot \left(N_{x}\alpha_{1}^{2}+N_{y}\alpha_{2}^{2}+N_{z}\alpha_{3}^{2}\right)$$

where *M*_s_ values were used from Table 3. Here, *N*_*x*_ and *N*_*z*_ are demagnetizing factors along the directions perpendicular (hard axis) and parallel (easy axis) to the fiber’s axis, respectively. The difference (*N*_*x*_–*N*_*z*_) quantifies the energy barrier originating from the geometric shape of the sample, reflecting the anisotropic nature of the demagnetizing fields due to the fiber’s elongated geometry. The demagnetizing factors (*N*_*x*_, *N*_*y*_, *N*_*z*_) were determined by modeling the rod-shaped sample as a prolate ellipsoid, in accordance with Osborn’s analytical formulation,^42^

The demagnetizing factors were calculated using the aspect ratio *m* = *c*/*a*, where *a* and *c* are the semiminor and semimajor axes of the ellipsoid, respectively

9 $$N_{x}=N_{y}=\frac{1-N_{z}}{2},N_{z}=\frac{1}{m^{2}-1}\left\{\frac{m}{\sqrt{m^{2}-1}}\ln\left(\frac{m+\sqrt{m^{2}-1}}{m-\sqrt{m^{2}-1}}\right)-1\right\}$$

To evaluate the impact of directional magnetic behavior from both theoretical and experimental perspectives, the calculated shape anisotropy energy (SAE) based on the Osborn ellipsoid model was compared to the second harmonic amplitude (*A*_2_) extracted from angular magnetization measurements as shown in Figure 9.

With increasing B_2_O_3_ addition, the normalized anisotropy relative to YIG systematically increases for SAE based on the theoretical Osborn model and *A*_2_ from Fourier fitting of experimental data from Figure 8c. This trend is broadly consistent with the *M*_s_^2^ scaling of SAE, since *M*_s_ also rises with B_2_O_3_ addition. However, the experimental *A*_2_ values grow less steeply than the SAE predictions which can be interpreted by considering the influence of geometric and microstructural factors. For in-plane rotation (α_3_ = 0), the SAE contains a cos2ϕ modulation with amplitude proportional to Δ*N* = *N*_*x*_ – *N*_*y*_. Any ellipticity (*N*_*x*_ ≠ *N*_*y*_) therefore enhances the *A*_2_ component. As shown in Figure 4, YIG fibers exhibit larger diameter fluctuations and higher defect densities, effectively increasing ellipticity. B_2_O_3_ addition suppresses such irregularities, stabilizing the molten zone and reducing geometry-driven deviations. In addition, slight uniaxial contributions arising from applied or internal stress, fixture misalignment, or processing-induced residual strain may also contribute to the observed *A*_2_ modulation. Taken together, ellipticity-driven demagnetization asymmetry and uniaxial effects explain why experimental *A*_2_ values deviate from the ideal *M*_s_^2^-scaled SAE, while still maintaining the overall upward trend with B_2_O_3_ doping.

Building upon the individual analyses of MCA and SAE, the total magnetic anisotropy energy is computed as their sum as

10 $$E_{total}=U_{MCA}+U_{SA}$$

and transformed into Cartesian coordinates for 3D visualization

11 $$X=E_{total}\times \sin\theta \times \cos\phi ,\;Y=E_{total}\times \sin\theta \times \sin\phi ,\;Z=E_{total}\times \cos\theta$$

The 3D surfaces reveal that the [111] direction coinciding with the fiber long axis is the EA with energy minima (blue regions), while the [100] and [010] directions align with HA near energy maxima (red regions). In Figure 10a, the MCA surfaces show that B_2_O_3_ doping results in a more anisotropic energy distribution than YIG, reflecting improved crystallographic alignment. The reference YIG single crystal exhibits the most pronounced anisotropy, with a minimum in energy at [111] and lobes along the HA, consistent with ideal single-crystal behavior.

In Figure 10c, the combined anisotropy energy surfaces show that the energy minima occur along the [111] direction consistent with the SA easy axis, also consistent with the MCA easy axis in cubic crystals. At this orientation, MCA contribution is minimized and further reduced by the SA component due to the sample geometry and demagnetizing effects.

The Faraday rotation of YIG and B_2_O_3_-assisted YIG fibers was measured using the optical setup shown in Figure 11a. A 1550 nm laser source was used to generate a linearly polarized optical beam, which was first passed through a fiber polarizer to ensure a well-defined initial polarization state. The collimated beam was then focused using a plano-convex lens and directed through the sample, which was positioned inside a ring magnet to apply a uniform axial magnetic field. The samples were mounted in a ceramic ferrule and polished on both sides before being placed inside the ring magnet, which generated an axial magnetic field of approximately 0.5 T which is saturated field for YIG samples to induce the Faraday effect.

**Figure 11:**
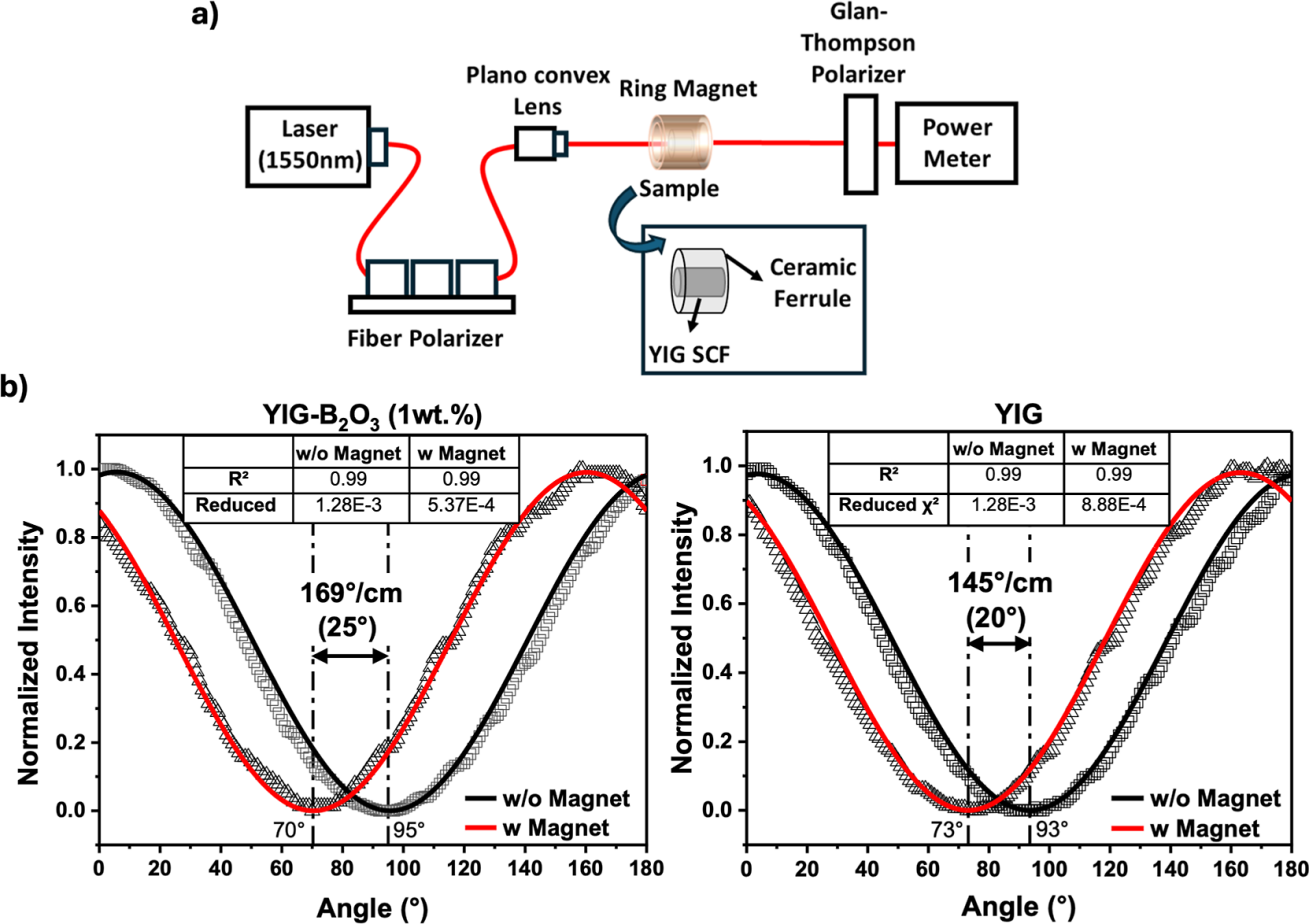
(a) Schematic of the experimental setup for Faraday rotation measurement (1550 nm). (b) Normalized intensity vs angle plots for YIG–B_2_O_3_ (1 wt %) and YIG measured with (red) and without (black) an applied magnetic field (0.5 T, saturated field).

The rotated polarization state was analyzed using a Glan-Thompson polarizer (extinction ratio ∼ 60 dB), and the transmitted intensity was measured using a power meter. To determine the Faraday rotation angle, the measurement was performed both with and without the ring magnet. The net Faraday rotation was determined using Malus’s Law fitting, which describes how the transmitted intensity (*I*) of a polarized beam changes as a function of the analyzer angle (θ)

12 $$I=I_{0}\cos^{2}\left(\theta -\Delta \theta \right)$$

where *I*_0_ is the maximum transmitted intensity, θ is the angle of the analyzer, and Δθ is the polarization shift induced by the Faraday effect. By fitting the intensity data to this equation, the shift in polarization between the magnetized and unmagnetized states (Δθ_F_) was extracted, corresponding to the Faraday rotation angle.

The specific Faraday rotation angles determined from the fits are 169°/cm for YIG–B_2_O_3_ (1 wt %) and 145°/cm for YIG, respectively. These values are consistent with reported literature values for undoped bulk YIG single crystals, which typically range from 160°/cm to 174°/cm at 1550 nm depending on the growth method (e.g., TSSG, FZ, SSFZ).^39,43,44^ The high *R* ^2^ values (0.99 for YIG-B_2_O_3_ (1 wt %) and 0.99 for YIG) and low reduced chi-square values (5.37 × 10^–4^ for YIG–B_2_O_3_(1 wt %) and 8.88 × 10^–4^ for YIG) confirm the strong agreement between the measured data and the Malus’s law fitting model.

The increased Faraday rotation in B_2_O_3_-assisted YIG fibers can be attributed to improved crystallographic orientation and purity of phases rather than a fundamental change in the Verdet constant. Unlike dopants that introduce additional electronic transitions or alter the intrinsic magnetization, B_2_O_3_ acts as a flux, promoting the growth of a more homogeneous YIG phase and suppressing secondary phase formation which reduces the net magnetization along the light propagation direction. A similar effect was reported for YIG grown via the Top Seeded Solution Growth (TSSG) method using B_2_O_3_–BaF_2_ flux, which exhibited a Faraday rotation of 160°/cm at 1550 nm.^39^ The close agreement between these values suggests that B_2_O_3_ primarily facilitates the fabrication of high-quality YIG single-crystal fiber without modifying the intrinsic magneto-optical properties of YIG. In contrast, dopants such as Ce^3+^ actively alter the electronic structure, introducing additional electronic transitions that significantly enhance the Verdet constant. For instance, fibrous YIG–Ce (0.6 at. %) grown by FZ method has been reported to exhibit ∼−1500°/cm at 1550 nm,^45^ attributed to altered magneto-optical transitions and strong spin–orbit coupling.^44^

## 4 Conclusions

This work demonstrates that B_2_O_3_ improves the laser heated pedestal growth (LHPG) of yttrium iron garnet (YIG) single-crystal fibers by lowering the solidus temperature and suggesting a reduced melt viscosity trend based on the TMA–VFT analysis. These effects contribute to more stable molten zone formation and improved fiber stability during growth. EBSD and SC-XRD analyses reveal improved crystallographic alignment along the [111] direction and a transition from polycrystalline to single crystal structure, without disrupting the garnet structure.

B_2_O_3_-assisted YIG fiber samples showed enhanced saturation magnetization, while maintaining coercivity, and remanent magnetization as YIG fibers without B_2_O_3_. Anisotropy analysis, incorporating EBSD-derived Euler angles into 3D energy surface calculations, highlights the modification in the directional distribution of the magnetocrystalline anisotropy energy due to improved crystallographic alignment.

The increasing dominant second harmonic observed in the angular magnetization analysis suggests a more pronounced expression of uniaxial shape anisotropy in B_2_O_3_-assisted YIG fibers. While crystallographic texture does not directly alter the shape anisotropy energy, improved [111] alignment and phase purity are expected to improve crystalline anisotropy by reducing local distortions and misoriented grains that enhance anisotropic response. The measured increase in Faraday rotation angle further supports enhanced magneto-optical behavior and uniformity.

Overall, B_2_O_3_-assisted LHPG growth shows the potential to yield high-quality YIG single crystal fibers with improved magnetic and magneto-optical properties, providing a pathway to scalable and nontoxic approaches suitable for integrated photonic and magnetic field sensing applications.

## Supplementary Material



## Notes

Supporting Information Schematic of the LHPG system; SEM surface morphology of sintered YIG and B_2_O_3_-doped YIG pellets; laser power vs fiber diameter variation during LHPG growth; EDX elemental mapping and line-scan composition distributions for YIG and YIG–B_2_O_3_ fibers; EDX maps and spectra of source pellets with quantitative composition results; temperature-dependent *M*–*T* curves with derivative analysis highlighting the Curie transition; expanded single-crystal XRD data collection and refinement statistics (PDF)

## References

[1] Princep A. J., Ewings R. A., Ward S., Tóth S., Dubs C., Prabhakaran D., Boothroyd A. T. (2017). The Full
Magnon Spectrum of Yttrium Iron Garnet. npj
Quantum Mater..

[2] Vojna D., Slezák O., Lucianetti A., Mocek T. (2019). Verdet Constant of
Magneto-Active Materials Developed for High-Power Faraday Devices. Appl. Sci..

[3] Wettling W., Andlauer B., Koidl P., Schneider J., Tolksdorf W. (1973). Optical Absorption and Faraday Rotation
in Yttrium
Iron Garnet. Phys. Status Solidi B Basic Res..

[4] Yokoi H., Mizumoto T., Shinjo N., Futakuchi N., Nakano Y. (2000). Demonstration of an Optical Isolator with a Semiconductor
Guiding Layer That Was Obtained by Use of a Nonreciprocal Phase Shift. Appl. Opt..

[5] Carter P. S. (1961). Magnetically-Tunable
Microwave Filters Using Single-Crystal Yttrium-Iron-Garnet Resonators. IRE Trans. Microwave Theory Tech..

[6] Huang M., Zheng W., Ji T., Ji M., Pu T., Qi B. (2024). Optical Sensor with Wide Range and High Sensitivity
for Internal
Magnetic Field Detection of Transformer. CSEE
J. Power Energy Syst..

[7] Sun L., Jiang S., Marciante J. R. (2010). All-Fiber Optical Magnetic-Field
Sensor Based on Faraday Rotation in Highly Terbium-Doped Fiber. Opt. Express.

[8] Srinivasan K., Stadler B. J. H. (2018). Magneto-Optical
Materials and Designs for Integrated
TE- and TM-Mode Planar Waveguide Isolators: A Review [Invited]. Opt. Mater. Express.

[9] Pirzada M., Altintas Z. (2020). Recent Progress in
Optical Sensors for Biomedical Diagnostics. Micromachines.

[10] Zhang J., Wang C., Chen Y., Xiang Y., Huang T., Shum P. P., Wu Z. (2022). Fiber Structures
and Material Science
in Optical Fiber Magnetic Field Sensors. Front.
Optoelectron..

[11] Fejer M. M., Nightingale J. L., Magel G. A., Byer R. L. (1984). Laser-Heated Miniature
Pedestal Growth Apparatus for Single-Crystal Optical Fibers. Rev. Sci. Instrum..

[12] Jundt D. H., Fejer M. M., Byer R. L. (1989). Growth
and Optical Properties of
Single-Crystal Sapphire Fibers. Proc. SPIE.

[13] Nie C. D., Bera S., Harrington J. A. (2016). Growth of Single-Crystal YAG Fiber
Optics. Opt. Express.

[14] Chen J.-C., Hu C.-C. (2003). Quantitative Analysis of YIG, YFeO3 and Fe3O4 in LHPG-Grown YIG Rods. J. Cryst. Growth.

[15] Lim H., DeMattei R. C., Feigelson R. S. (1997). Growth
of Single Crystal Yig Fibers
by the Laser Heated Pedestal Growth Method. MRS Online Proc. Libr..

[16] Kimura S., Shindo I. (1977). Single Crystal Growth
of YIG by the Floating Zone Method. J. Cryst.
Growth.

[17] Mao T.-C., Chen J.-C., Hu C.-C. (2005). Characterization
of the Growth Mechanism
of YIG Crystal Fibers Using the Laser Heated Pedestal Growth Method. J. Cryst. Growth.

[18] Tolksdorf W. (1977). Growth of
Magnetic Garnet Single Crystals from High Temperature Solution. J. Cryst. Growth.

[19] Antonini B., Paoletti A., Paroli P. (1981). Isothermal
Growth of Bulk Yig Crystals
by PbF2-B2O3 Flux Evaporation. J. Cryst. Growth.

[20] Linares R. C. (1962). Growth
of Yttrium-Iron Garnet from Molten Barium Borate. J. Am. Ceram. Soc..

[21] Bandyopadhyay T., Saha P. (1977). Growth of Yttrium Iron
Garnet Single Crystals in Na2O-B2O3 Flux System
in Air. Pramana.

[22] Kučera M., Nitsch K., Štěpánková H., Maryško M., Reiche P. (2003). Growth and Characterization of High
Purity Epitaxial Yttrium Iron Garnet Films Grown from BaO–B2O3–BaF2
Flux. Phys. Status Solidi.

[23] Jonker H. D. (1975). Investigation
of the Phase Diagram of the System PbO-B2O3-Fe2O3-Y2O3 for the Growth
of Single Crystals of Y3Fe5O12. J. Cryst. Growth.

[24] Andlauer B., Tolksdorf W. (1974). Local Vibrational
Modes of Boron in Garnets. Phys. Status Solidi
B Basic Res..

[25] Andlauer B., Tolksdorf W. (1979). Associated
Incorporation of Boron, Lead, and Oxygen
Vacancies in Garnets. J. Appl. Phys..

[26] Karki, D. ; Hoffman, E. ; Shengye, D. ; Schmotzer, V. T. ; Liu, B. ; Ohodnicki, P. R. Machine Vision Approach of Process Control during Single Crystal Fiber Growth via Laser Heated Pedestal Growth Method. In Fiber Optic Sensors and Applications XVIII; Sanders, G. A. , Lieberman, R. A. , Scheel, I. U. , Eds., 2022; p 13. Eds.; SPIE: Orlando, United States. 10.1117/12.2618538.

[27] Koštál P., Hofírek T., Málek J. (2018). Viscosity Measurement by Thermomechanical
Analyzer. J. Non-Cryst. Solids.

[28] Fontana E. H. (1970). A Versatile
Parallel-Plate Viscometer for Glass Viscosity Measurements to 1000
°C. Am. Ceram. Soc. Bull..

[29] Sinn E. (1979). Measurement
of the Shear Viscosity in Garnet High Temperature Solutions by a Cylindrical
Rotation Viscometer. Krist. Tech..

[30] Lim H.-J., DeMattei R. C., Feigelson R. S., Rochford K. (2000). Striations in YIG Fibers
Grown by the Laser-Heated Pedestal Method. J.
Cryst. Growth.

[31] Maljuk A., Watauchi S., Tanaka I., Kojima H. (2000). The Effect of B2O3
Addition on La2-xSrxCuO4 Single-Crystal Growth. J. Cryst. Growth.

[32] Goya G. F., Berquó T. S., Fonseca F. C., Morales M. P. (2003). Static and Dynamic
Magnetic Properties of Spherical Magnetite Nanoparticles. J. Appl. Phys..

[33] Shang M., Zhang C., Zhang T., Yuan L., Ge L., Yuan H., Feng S. (2013). The Multiferroic
Perovskite YFeO3. Appl. Phys. Lett..

[34] Aleksandrov K. S., Bezmaternykh L. N., Kozlov G. V., Levedev S. P., Mukhin A. A., Prokhorov A. S. (1987). Anomalies of High-Frequency Magnetic Permeability of
Hematite at the Morin Phase Transition. J. Exp.
Theor. Phys..

[35] Tolksdorf W., Tolle H. J., Klages C. P. (1982). SIMS Analysis
of Lead and Boron in
Yttrium Iron Garnet Epilayers. J. Cryst. Growth.

[36] Milanese C., Buscaglia V., Maglia F., Anselmi-Tamburini U. (2004). Disorder and
Nonstoichiometry in Synthetic Garnets A_3_ B_5_ O_12_ (A = Y, Lu–La, B = Al, Fe, Ga). A Simulation Study. Chem. Mater..

[37] Müller, P. ; Herbst-Irmer, R. ; Spek, A. L. ; Schneider, T. R. ; Sawaya, M. R. Crystal Structure Refinement: A Crystallographer’s Guide to SHELXL, 1 ed.; Oxford University Press: Oxford, 2006; . st ed.10.1093/acprof:oso/9780198570769.001.0001.

[38] Sánchez R. D., Rivas J., Vaqueiro P., López-Quintela M. A., Caeiro D. (2002). Particle Size Effects on Magnetic Properties of Yttrium
Iron Garnets Prepared by a Sol–Gel Method. J. Magn. Magn. Mater..

[39] Yang X., Lan J., Wei Z., Su R., Li Y., Wang Z., Liu Y., He C., Long X. (2023). High Quality and Large Size Yttrium
Iron Garnet Crystal Grown by Top Seeded Solution Growth Technique. J. Inorg. Mater..

[40] Bunge, H.-J. 12 - Orientation Distribution Functions of Other Structural Elements. In Texture Analysis in Materials Science; Bunge, H.-J. , Ed.; Butterworth-Heinemann, 1982; pp 279–293.10.1016/B978-0-408-10642-9.50017-9.

[41] Hansen P. (1974). Anisotropy
and Magnetostriction of Gallium-Substituted Yttrium Iron Garnet. J. Appl. Phys..

[42] Osborn J.
A. (1945). Demagnetizing
Factors of the General Ellipsoid. Phys. Rev..

[43] Sekijima T., Satoh H., Tahara K., Fujii T., Wakino K., Okada M. (1998). Growth of Fibrous YIG Single Crystals
by the Self-Adjusting Solvent
FZ Method. J. Cryst. Growth.

[44] Ikesue A., Aung Y. L., Yasuhara R., Iwamoto Y. (2020). Giant Faraday
Rotation
in Heavily Ce-Doped YIG Bulk Ceramics. J. Eur.
Ceram. Soc..

[45] Sekijima T., Itoh H., Fujii T., Wakino K., Okada M. (2001). Influence
of Growth Atmosphere on Solubility Limit of Ce3+ Ions in Ce-Substituted
Fibrous Yttrium Iron Garnet Single Crystals. J. Cryst. Growth.

