# Supplementary material for: Effects of B2O3 on the Growth, Structural, and Magneto-Optical Properties of Yttrium Iron Garnet Single-Crystal Fibers
Source: Cryst Growth Des. 2026 Mar 4;26(6):2473–87. doi: 10.1021/acs.cgd.5c01776 (PMC13003439; doi:10.1021/acs.cgd.5c01776)
Supplement: Supplementary file 1 [file cg5c01776_si_001.pdf]

Supporting Information

Effects of B_2O_3 on the Growth, Structural, and Magneto-Optical Properties of Yttrium Iron Garnet Single-Crystal Fibers

Jun Young Hong [1], Dolendra Karki [1,4], Soumya Sridar [1], Paul Ohodnicki [1,2,3*]

[1)] Department of Mechanical & Materials Science and Engineering, University of Pittsburgh, Pittsburgh, PA, 15261, USA

[2)] Department of Electrical and Computer Engineering, University of Pittsburgh, Pittsburgh, PA, 15261, USA

[3)] Department of Physics and Astronomy, University of Pittsburgh, Pittsburgh, PA, 15261, USA

[4)] National Energy Technology Laboratory, Morgantown, West Virginia 26505, USA

*Corresponding author: pro8@pitt.edu

Table of Contents

Figure S1. Schematic of LHPG system

Figure S2. SEM images of the surface morphology of sintered YIG and B_2O_3-doped YIG pellets

Figure S3. Correlation between laser power (right y-axis, W) and diameter variation (left y-axis, μm) during the LHPG growth of YIG and YIG-B_2O_3 (0.5 and 1 wt.%) crystals

Figure S4. EDX analysis of YIG and YIG-B_2O_3(1 wt.%) crystals: (a) elemental mapping of Y, Fe, O, and B on sample surfaces and (b) distribution of elemental composition (at.%) from multiple EDX line scans along both the vertical or horizontal direction relative to the growth axis

Figure S5. EDX maps and spectra of the surfaces of source pellets (undoped, 0.5, 1, and 5 wt.% B_2O_3) prior to LHPG growth.

Figure S6. Temperature dependence of magnetization (M) for (a) YIG, (b) YIG–B_2O_3(0.5 wt.%), and (c) YIG–B_2O_3(1 wt.%) fibers. Insets show the corresponding derivative curves (dM/dT), where the Curie transition of YIG near 555 K is highlighted. The full width at half maximum (FWHM) and the minimum derivative value (dM/dTmin) at 555K are indicated for this transition

Table S1. EDX quantification results (wt.%) for the surfaces of YIG and B_2O_3-doped YIG source pellets used in LHPG growth. Values represent mean compositions from area scans; σ denotes standard deviation from multiple measurement regions.

Table S2. Expanded single-crystal XRD data collection and refinement statistics for YIG and YIG–B_2O_3 samples.

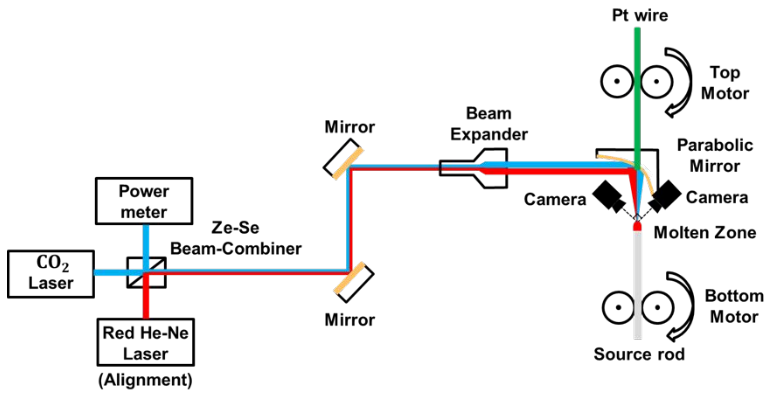

Figure S1. Schematic of LHPG system

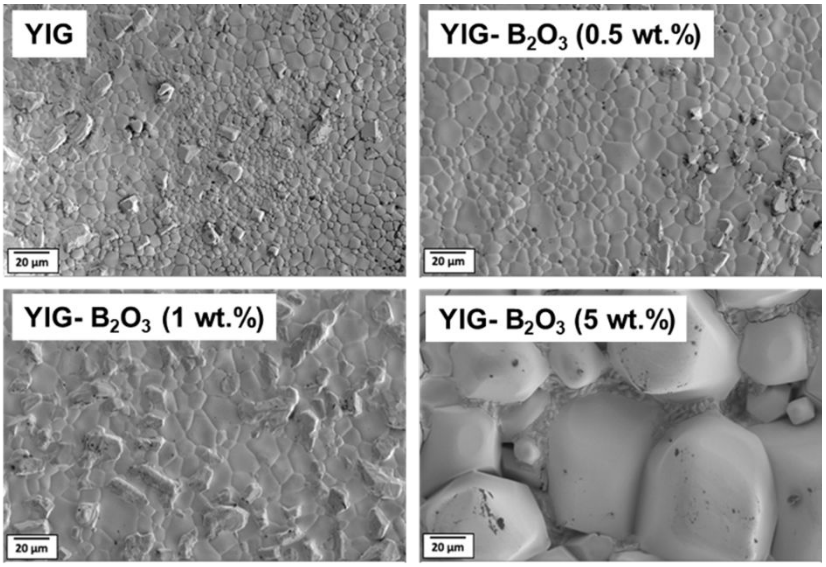

Figure S2. SEM images of the surface morphology of sintered YIG and B_2O_3-doped YIG pellets

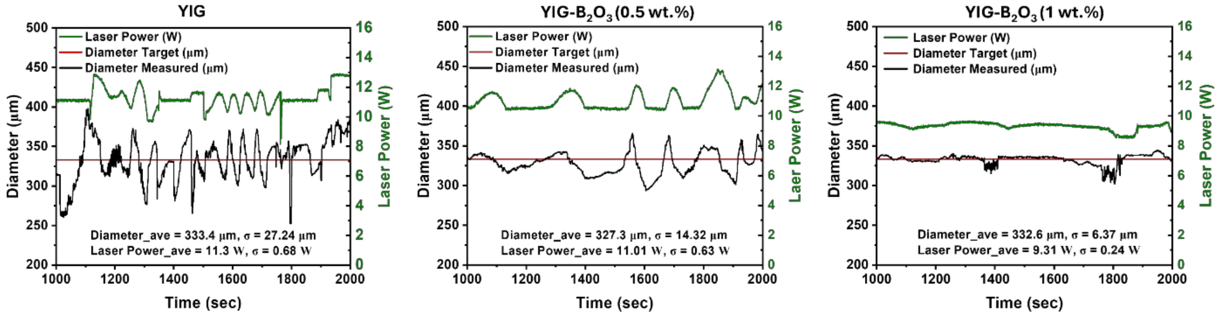

Figure S3. Correlation between laser power (right y-axis, W) and diameter variation (left y-axis, µm) during the LHPG growth of YIG and YIG-B_2O_3 (0.5 and 1 wt.%) crystals

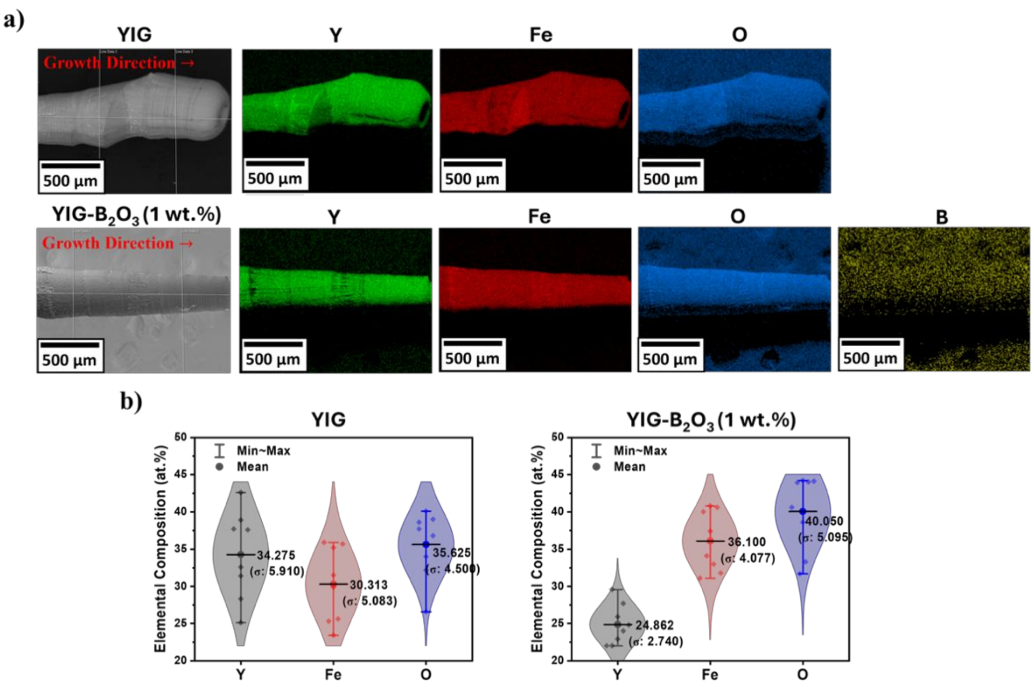

Figure S4. EDX analysis of YIG and YIG-B_2O_3(1 wt.%) crystals: (a) elemental mapping of Y, Fe, O, and B on sample surfaces and (b) distribution of elemental composition (at.%) from multiple EDX line scans along both the vertical or horizontal direction relative to the growth axis

In Figure S4b, violin plots represent the distribution of elemental compositions (at.%) based on multiple EDX scans. Each plot includes the mean value, min-max range, and standard deviation (σ) to represent both central tendency and satistical spread. For YIG, the mean values for Y, Fe, and O are 34.275%, 30.313%, and 35.625%, respectively, with standard deviations (σ) of 5.910%, 5.083%, and 4.500%. These values reflect higher variability in the elemental composition, particularly for Y and Fe, which aligns with the observed surface segregation in Figure S4a. In contrast, for the B_2O_3-assisted YIG sample, the mean values for Y, Fe, and O are 24.862%, 36.100%, and 40.050%, with smaller standard deviations of 2.740%, 4.077%, and 5.095%, respectively. While oxygen (O) measurements in EDX can be unreliable, the B_2O_3-assisted sample shows an overall improved Y:Fe atomic ratio of approximately 0.689:1, which is closer to the ideal stoichiometric ratio of 0.6:1 (3:5) compared to the YIG sample with a ratio of 1.13:1. This reduced variability, particularly for Y, indicates enhanced compositional uniformity and suggests improved alignment with the stoichiometric YIG phase. The normal distribution curves overlaid on the violin plots further confirm that the B_2O_3-assisted YIG exhibits narrower spreads and better alignment of elemental compositions around the mean. This indicates improved phase homogeneity compared to the YIG sample.

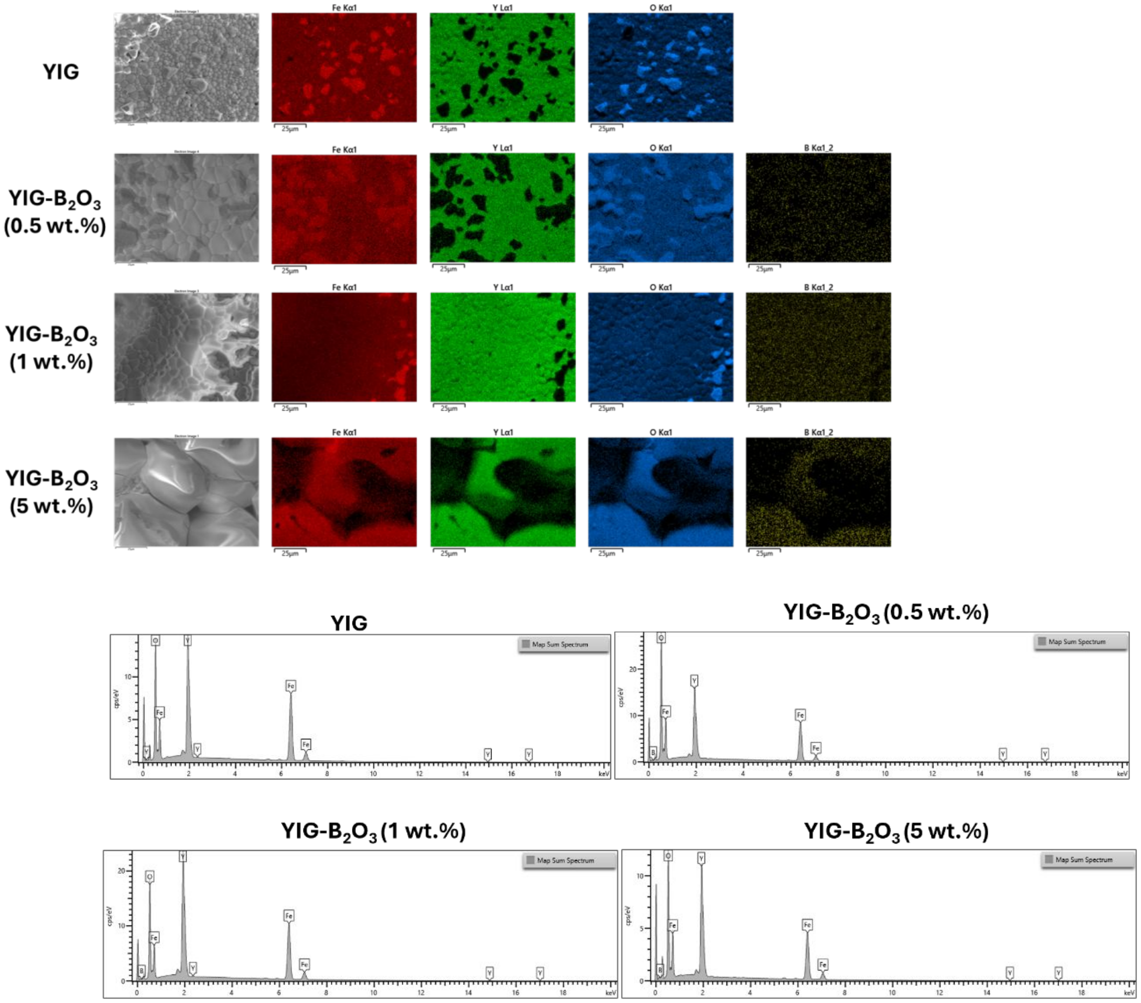

Figure S5. EDX maps and spectra of the surfaces of source pellets (undoped, 0.5, 1, and 5 wt.% B_2O_3) prior to LHPG growth.

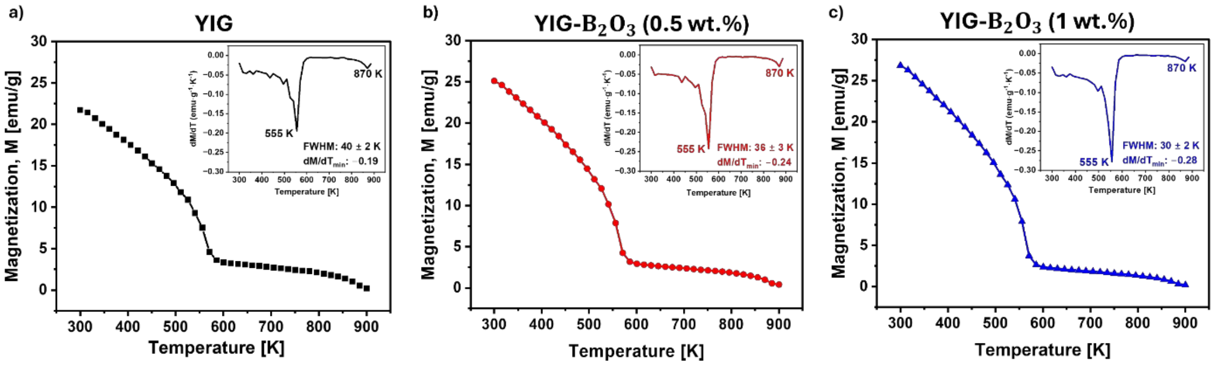

Figure S6. Temperature dependence of magnetization (M) for (a) YIG, (b) YIG–B_2O_3(0.5 wt.%), and (c) YIG–B_2O_3(1 wt.%) fibers. Insets show the corresponding derivative curves (dM/dT), where the Curie transition of YIG near 555 K is highlighted. The full width at half maximum (FWHM) and the minimum derivative value (dM/dTmin) at 555K are indicated for this transition

High-temperature M–T measurements (300–900 K) with the magnetic field applied parallel to the fiber axis are shown in Figure 10. The Curie transition near 555 K was evaluated using the full width at half maximum (FWHM) of the dM/dT dip, which reflects the spread of transition temperatures, and its minimum value (dip depth).

In Figure S6 (a), the YIG fiber shows a broader transition (FWHM ≈ 40 ± 2 K, dip depth ≈ –0.19 $emu \cdot g^{-1} \cdot K^{-1}$) at 555K and an additional anomaly near 870 K attributable to Fe_3O_4 [33], consistent with secondary phases seen in EBSD (Figure 5). By contrast, B_2O_3-assisted fibers exhibit sharper transitions (FWHM ≈ 36 ± 3 K, dip depth ≈ –0.24 $emu \cdot g^{-1} \cdot K^{-1}$ for 0.5 wt.% and FWHM ≈ 30 ± 2 K, dip depth ≈ –0.28 $emu \cdot g^{-1} \cdot K^{-1}$ for 1 wt.%) at 555K, indicating improved homogeneity [33]. The suppression of the 870 K feature further confirms reduced Fe_3O_4 contributions, consistent with enhanced YIG phase purity.

Table S1. EDX quantification results (wt.%) for the surfaces of YIG and B_2O_3-doped YIG source pellets used in LHPG growth. Values represent mean compositions from area scans; σ denotes standard deviation from multiple measurement regions.

Sample	Fe		Y		O		B	
	wt.%	σ	wt.%	σ	wt.%	σ	wt.%	σ
YIG	31.44	0.06	33.12	0.07	35.45	0.07	N/A	N/A
YIG-B_2O_3 (0.5 wt.%)	26.4	0.23	27.62	0.25	45.5	0.37	0.48	0.75
YIG-B_2O_3 (1 wt.%)	28.68	0.09	36.47	0.11	33.94	0.1	0.92	0.25
YIG-B_2O_3 (5 wt.%)	24.31	0.23	31.91	0.3	39.52	0.36	4.26	0.8

Table S2. Expanded single-crystal XRD data collection and refinement statistics for YIG and YIG–B_2O_3 samples.

Parameter	YIG	YIG-B_2O_3 (0.5 wt%)	YIG-B_2O_3 (1 wt%)
Temperature (K)	300		
Radiation, λ (Å)	Mo Kα (0.71073 Å)		
Diffractometer	Bruker D8 Venture (IμS 3.0)		
θ range (°)	4.04–28.31	4.04–28.29	3.29–28.69
Reflections collected	19803	23429	27232
Independent reflections	6010	7029	8169
R_{int}	0.0543	0.0481	0.0432
Absorption correction	Multi-Scan method (SADABS)		
Transmission ratio (min/max)	0.250	0.236	0.245
Refinement method	Full-matrix least-squares on F^2		
GooF on F^2	1.453	1.234	0.934
R1, wR2 [I>2σ(I)]	0.153, 0.265	0.042, 0.113	0.031, 0.107
R1, wR2 (all data)	0.184, 0.284	0.050, 0.135	0.040, 0.137
Largest diff peak/hole ($e^- Å^{-3}$)	1.345 / -1.574	1.745 / -1.634	1.981 / -1.462
